# The antiviral sirtuin 3 bridges protein acetylation to mitochondrial integrity and metabolism during human cytomegalovirus infection

**DOI:** 10.1371/journal.ppat.1009506

**Published:** 2021-04-15

**Authors:** Xinlei Sheng, Ileana M. Cristea

**Affiliations:** Department of Molecular Biology, Princeton University, Lewis Thomas Laboratory, Princeton, NJ, United States of America; State University of New York Upstate Medical University, UNITED STATES

## Abstract

Regulation of mitochondrial structure and function is a central component of infection with viruses, including human cytomegalovirus (HCMV), as a virus means to modulate cellular metabolism and immune responses. Here, we link the activity of the mitochondrial deacetylase SIRT3 and global mitochondrial acetylation status to host antiviral responses via regulation of both mitochondrial structural integrity and metabolism during HCMV infection. We establish that SIRT3 deacetylase activity is necessary for suppressing virus production, and that SIRT3 maintains mitochondrial pH and membrane potential during infection. By defining the temporal dynamics of SIRT3-substrate interactions during infection, and overlaying acetylome and proteome information, we find altered SIRT3 associations with the mitochondrial fusion factor OPA1 and acetyl-CoA acyltransferase 2 (ACAA2), concomitant with changes in their acetylation levels. Using mutagenesis, microscopy, and virology assays, we determine OPA1 regulates mitochondrial morphology of infected cells and inhibits HCMV production. OPA1 acetylation status modulates these functions, and we establish K834 as a site regulated by SIRT3. Control of SIRT3 protein levels or enzymatic activity is sufficient for regulating mitochondrial filamentous structure. Lastly, we establish a virus restriction function for ACAA2, an enzyme involved in fatty acid beta-oxidation. Altogether, we highlight SIRT3 activity as a regulatory hub for mitochondrial acetylation and morphology during HCMV infection and point to global acetylation as a reflection of mitochondrial health.

## Author summary

Given their functions in cellular metabolism and immune responses, mitochondria are targeted and disrupted by numerous prevalent viral pathogens, including human cytomegalovirus (HCMV). To characterize mechanisms underlying mitochondrial regulation during HCMV infection in human fibroblasts, this study integrates enzyme-substrate interaction studies, mass spectrometry quantification of protein abundance and acetylation, mutagenesis, microscopy, and virology assays. These analyses establish a link between the mitochondrial acetylation status and mitochondrial structure and metabolism during HCMV infection. We demonstrate that the mitochondrial deacetylase SIRT3 acts in host defense by modulating proteins that regulate mitochondrial structure and fatty acid oxidation. SIRT3 helps to maintain mitochondrial integrity through several mechanisms, including regulation of mitochondrial pH, membrane potential, and the balance between mitochondrial fission and fusion. As excess mitochondrial acetylation is detrimental to mitochondrial metabolism, the virus-induced alterations in SIRT3 functions and mitochondrial acetylation may be linked to known HCMV pathologies, such as the metabolic syndrome and cardiac hypertrophy.

## Introduction

The progression of a viral infection and production of virus progeny within host cells rely on the spatial and temporal modulation of cellular processes. Systematic structural and functional remodeling of cellular organelles is a common feature at the core of the virus replication cycles for numerous DNA and RNA viruses [1]. Given its functions in metabolism, apoptosis and immune signaling, the mitochondrion is the target of diverse viruses, including but not limited to *Herpesviridae* [2,3], *Retroviridae* [4,5], *Flaviviridae* [6,7], and *Orthomyxoviridae* [8,9]. Among herpesviruses, human cytomegalovirus (HCMV) is a β-herpesvirus that is prevalent in the population and that leads to congenital diseases and severe pathologies in immunocompromised individuals [10,11]. One hallmark of HCMV infection is the disruption of mitochondrial morphology and metabolism. These virus-induced alterations are aimed at inhibiting cellular defenses and providing the energy and metabolites necessary for the production of infectious viral particles [12,13].

Evident upon HCMV infection is the enhanced fragmentation of the mitochondrial network [14]. In uninfected cells, mitochondria display extended shapes, forming large filamentous networks [15]. Upon HCMV infection, the antiapoptotic viral protein pUL37x1 (also known as the viral mitochondrion-localized inhibitor of apoptosis, vMIA) [16] targets the mitochondria and promotes fission [14] by inducing the release of Ca^2+^ from the ER into the cytosol [17], which subsequently elevates Ca^2+^ levels in the mitochondria [18]. As pUL37x1 is an immediate early viral protein, the onset of mitochondrial fission occurs at an early stage of infection when viral genomes are starting to be replicated. During the progression of infection, mitochondria undergo changes in their subcellular localization, by late in infection becoming distributed around the newly formed viral assembly complex [19]. HCMV also alters the mitochondrial metabolism, promoting ATP synthesis and fatty acid synthesis. Elevated fatty acid levels are needed to support virus envelopment during virion assembly late in infection [20].

At the core of these structural and metabolic mitochondrial manipulations are changes in organelle composition, with mitochondrial transcription and translation being upregulated during infection [21]. Outside the context of infection, accumulating evidences are also pointing to the contribution of protein posttranslational modifications (PTMs) to the regulation of mitochondria functions and morphology. Mitochondrial protein phosphorylation, acetylation, SUMOylation, ubiquitination, malonylation and lipoylation, among others, were shown to modulate the balance between mitochondrial fission and fusion, as well as the function of mitochondrial metabolic protein complexes [15,22,23]. For example, both SUMOylation and phosphorylation of the fission factor GTPase dynamin-related protein 1 (DRP1) were shown to regulate mitochondrial fission [24,25]. Additionally, the acetylation of the fusion factor, optic atrophy 1 (OPA1) [26], was shown to inhibit its activity and disrupt the formation of mitochondrial reticular networks [27].

Among these mitochondrial PTMs, protein lysine acetylation came into the spotlight in the context of HCMV infection [28], as we discovered that the mitochondrial acetylome is globally elevated during the progression of HCMV infection [29]. The range of modified mitochondrial proteins agrees with the growing understanding in uninfected cells that acetylation can be prevalent within mitochondria, with over 60% of the proteins with mitochondrial localization reported to have acetylated lysines [30]. Since the functions for the majority of these acetylations are unknown, it remains to be determined whether these modifications can impact mitochondrial functions during HCMV infection. Suggesting that elevated mitochondrial acetylation may benefit the virus, we reported that the mitochondrial deacetylase sirtuin 3 (SIRT3) [31] acts in antiviral response against HCMV [32]. However, it remains to be demonstrated whether this SIRT3 defense function relies on its deacetylase activity.

Studies in Sirt3 knockout mice, as well as mouse and human cells, predicted that SIRT3 has over a hundred mitochondrial substrates [33–37]. As indicated above, most of these acetylation sites remain to be functionally characterized. However, accumulating evidences support a role for acetylation in enzyme activities, protein interactions, protein subcellular localization, and protein abundance (e.g., by preventing the deposition of ubiquitination at specific lysine residues). In uninfected cells, SIRT3-mediated deacetylation events have been linked to the maintenance of mitochondrial integrity. Upon loss of mitochondrial membrane potential, SIRT3 was reported to dissociate from the inner mitochondrial membrane and to gain interactions with mitochondrial matrix proteins [38]. SIRT3 deacetylation of OPA1 K931 promoted filamentous mitochondrial structure in response to stress [27]. These observations indicate that SIRT3 can respond to external perturbations and regulate mitochondrial integrity via modulation of its substrates. Additionally, mitochondrial metabolic proteins have also been identified or predicted as SIRT3 substrates. For example, proteins involved in fatty acid metabolism, such as AceCS2, VLCAD, and LCAD, were shown to be activated by SIRT3 [39–41]. SIRT3 also activates the superoxide dismutase SOD2 by deacetylation, alleviating the reactive oxygen species (ROS) during stress [42]. Considering the global disruption of mitochondria during HCMV infection and the breadth of SIRT3 substrates, it is tempting to propose that SIRT3 executes its antiviral functions via its deacetylation activity and its regulation of both mitochondrial integrity and metabolism. More broadly, it is possible that the global mitochondrial acetylation status provides a representation of the structure and metabolic function of this organelle.

Here, we investigate the link between SIRT3 function, protein acetylation, and mitochondrial integrity during HCMV infection. We validate the antiviral function of SIRT3, and demonstrate the requirement of its deacetylase activity for this host defense function. We show that the SIRT3 activity helps to maintain the mitochondrial membrane potential and the mitochondrial pH during infection. Using immunoaffinity purification and mass spectrometry (IP-MS), we characterize the temporal dynamics of SIRT3-substrate interactions during the progression of HCMV infection. Integrating temporal acetylation data, microscopy, and functional assays, we establish OPA1 as a virus restriction factor and demonstrate the functional regulatory abilities of acetylation at K834 and K931. We show SIRT3 enzymatic activity is necessary for removing OPA1 K834 acetylation in cells, a residue not previously known to be regulated by SIRT3. Additionally, SIRT3-OPA1 interaction is increased early in infection, but then disrupted at the later stage, in conjunction with a matching decrease and then increase in K834 acetylation. This suggests that the host cell responds in defense by promoting SIRT3-mediated OPA1 deacetylation, and this is then counteracted by the virus later in infection. We further demonstrate that the SIRT3 overexpression or knockdown induces mitochondrial morphological changes, which broadly links the mitochondrion acetylation status to its structure. Lastly, we show that temporal alterations in SIRT3-substrate interactions may also underlie HCMV-induced changes in fatty acid metabolism. The SIRT3 interaction with acetyl-CoA acyltransferase 2 (ACAA2) [35], a putative substrate involved in fatty acid beta-oxidation, is lost upon infection, leading to elevated ACAA2 acetylation levels. We further determine that ACAA2 can normally inhibit HCMV production, suggesting that this change in acetylation status likely inhibits its function and benefits virus production. Altogether, our study highlights the deacetylase activity of SIRT3 as a regulatory hub that can be used to broadly control mitochondrial acetylation, integrity, and metabolism during HCMV infection.

## Results

### Mitochondrial protein acetylation is globally upregulated during HCMV infection

**Fig 1:**
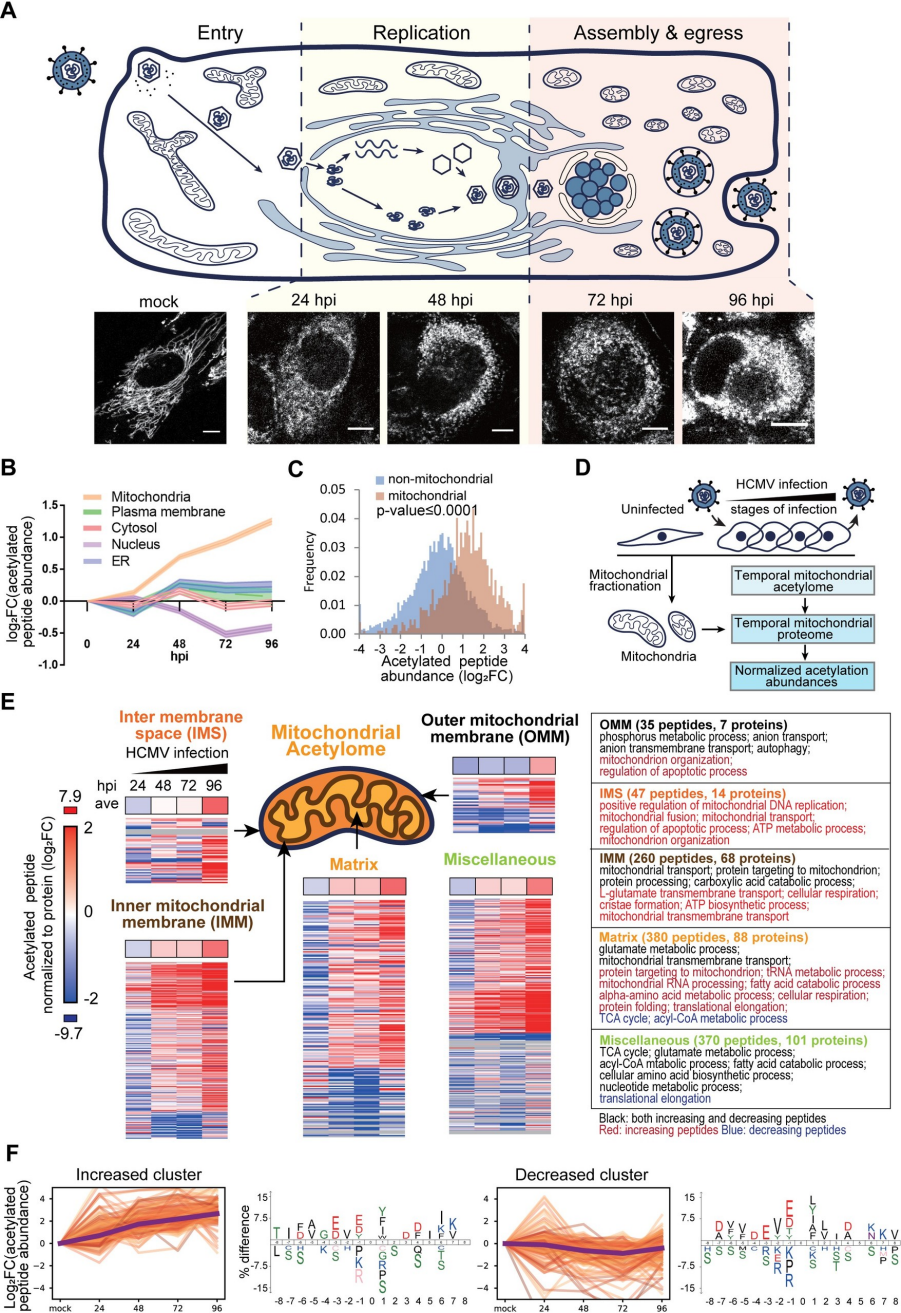
HCMV infection elevates acetylation levels in mitochondria. A. Representation of the replication cycle of HCMV (upper) and mitochondrial fragmentation during infection depicted by fluorescence microscopy (Mitotracker Red CMXROS as mitochondria marker) in primary human fibroblasts (lower). hpi: hours post infection. Scale bar: 10 μm. B. Changes in average relative peptide acetylation levels in different subcellular localizations, including mitochondria, plasma membrane, cytosol, nucleus, and ER. The average abundances of the acetylated peptides (without normalization to protein abundances; normalized abundances are shown in S1A Fig) at different time points of infection are shown as normalized to levels in mock cells ± SE. C. Frequency density of acetylated peptides abundances (log2 fold-change, not normalized to protein abundances; normalized levels are shown in S1B Fig) in the mitochondria and non-mitochondrial localizations at 120 hpi. Mann Whitney U test, p-value <0.0001. D. Schematic workflow for analyzing the temporal mitochondrial acetylome and proteome during HCMV infection. Uninfected and infected human fibroblasts were collected at multiple time points, and the mitochondria were enriched using density fractionation. Temporal mitochondrial proteome was analyzed by LC-MS/MS, and then integrated with the temporal mitochondrial acetylome dataset to derive normalized acetylation abundances. E. Changes in peptide acetylation abundances within different mitochondrial compartments, i.e. Outer mitochondrial membrane (OMM), Inter membrane space (IMS), Inner mitochondrial membrane (IMM), matrix, and miscellaneous. The averaged abundances (bar at the top) and site-specific abundances of the acetylated peptides (bottom) are represented for each compartment (log2FC). Enriched GO biological processes are shown for proteins with increasing (red) and decreasing (blue) acetylation trends. Black indicates terms shared by both increasing and decreasing trends. F. Representative fuzzy-c means clusters of acetylated peptide abundances during infection and their corresponding motifs. Fuzzy-c means clustering was conducted on log2 fold change of acetylated peptide abundance normalized to mock. The increasing and decreasing clusters are selected, and their consensus sequences were generated using IceLogo.

HCMV is known to induce alterations in mitochondrial structure and function during the progression of its replication cycle [14,17,19,43–45] (Fig 1A, *top*). These changes are aimed at modulating cellular metabolism and suppressing apoptosis and immune responses [14,46–48], among other, and are accompanied by an evident increase in mitochondrial fragmentation (Fig 1A, *bottom*). From our previous study of the whole-cell protein acetylome during HCMV infection [29], we identified approximately 990 acetylated peptides from mitochondrial proteins across infection time points. We now revisited this global acetylome dataset and analyzed the temporality of these mitochondrial acetylation events. We plotted the abundance of the acetylated peptides during the HCMV replication cycle (24, 48, 72 and 96 hours post infection (hpi)) normalized to uninfected cells (mock). We noticed that infection results in a global increase in the acetylation of mitochondrial proteins to a higher level than of proteins with other subcellular localizations, including plasma membrane, cytosol, ER and nucleus (Fig 1B). This upregulation is not driven by a subset of peptides, as we observed a global shift in abundances compared to non-mitochondria acetylated peptides (Fig 1C). Notably, this alteration is not primarily driven by protein abundances, as the global upregulation is maintained after normalization to infection-driven changes in protein abundances (S1A and S1B Fig). Therefore, we asked whether this prominent change in acetylation status is connected to the HCMV regulation of mitochondrial function and induction of mitochondrial fragmentation. To address this question, we started by defining more accurately these acetylation changes, then investigating their regulation and the functions of site-specific acetylation events.

To more accurately measure alterations in acetylation, we analyzed temporal changes in mitochondrial protein abundances during infection (Fig 1D). Fibroblasts were collected either as uninfected or following infection at time points representing different stages of HCMV replication, and the mitochondria were enriched by density gradient ultracentrifugation. Mitochondrial protein abundances were relatively quantified by label-free MS (S1C Fig and S1 Table) and then used to normalize the abundances of acetylated peptides. Overall increasing trends in acetylation levels were observed for proteins with known localization to the inner mitochondrial membrane (IMM), inter-membrane space (IMS), and matrix (Fig 1E and S2 Table). Many outer mitochondrial membrane (OMM) proteins displayed decreased acetylation levels at 24, 48 and 72 hpi, and although increasing at 96 hpi, the average acetylation abundance was still lower compared to other sub-mitochondrial regions. GO enrichment analysis indicated that infection induces increased acetylation levels on proteins in several key mitochondrial metabolic pathways, including fatty acid beta-oxidation, electron transport chain and regulation of proteolysis (shown for 96 hpi in Fig 1E, *right*).

Analysis of the amino acids flanking the identified acetylation sites (S1D Fig) showed consensus motifs that partially agree with reports of mitochondrial proteins in uninfected cells [33–35,49], as well as of bacterial proteins [50]. Amino acids negatively charged or with small side chains were observed preceding the acetylated lysines; hydrophobic residues (Y and F) were found at +1 and +2 positions, whereas lysines were enriched at +6. This enrichment pattern resembles that reported for non-enzymatic acetylation [51,52]. To determine whether other motifs can be specifically assigned to the subsets of increasing or decreasing acetylation sites, we performed fuzzy-c means clustering based on temporal acetylation trends (Figs 1F and S1E). The motifs observed for the increasing and decreasing acetylation clusters were similar to the global mitochondria acetylome motifs and remained consistent throughout the virus replication cycle. Altogether, our finding of a broad upregulation of mitochondrial protein acetylation and the observed consensus motif indicate the likely presence of non-enzymatic acetylation during infection, which is in agreement with the absence of a known prominent mitochondrial acetyltransferase and previous metabolomic studies that reported increased acetyl-CoA levels following HCMV infection [20,53,54].

### The SIRT3 deacetylase activity contributes to its antiviral function, and SIRT3 maintains mitochondrial membrane potential and pH during infection

**Fig 2:**
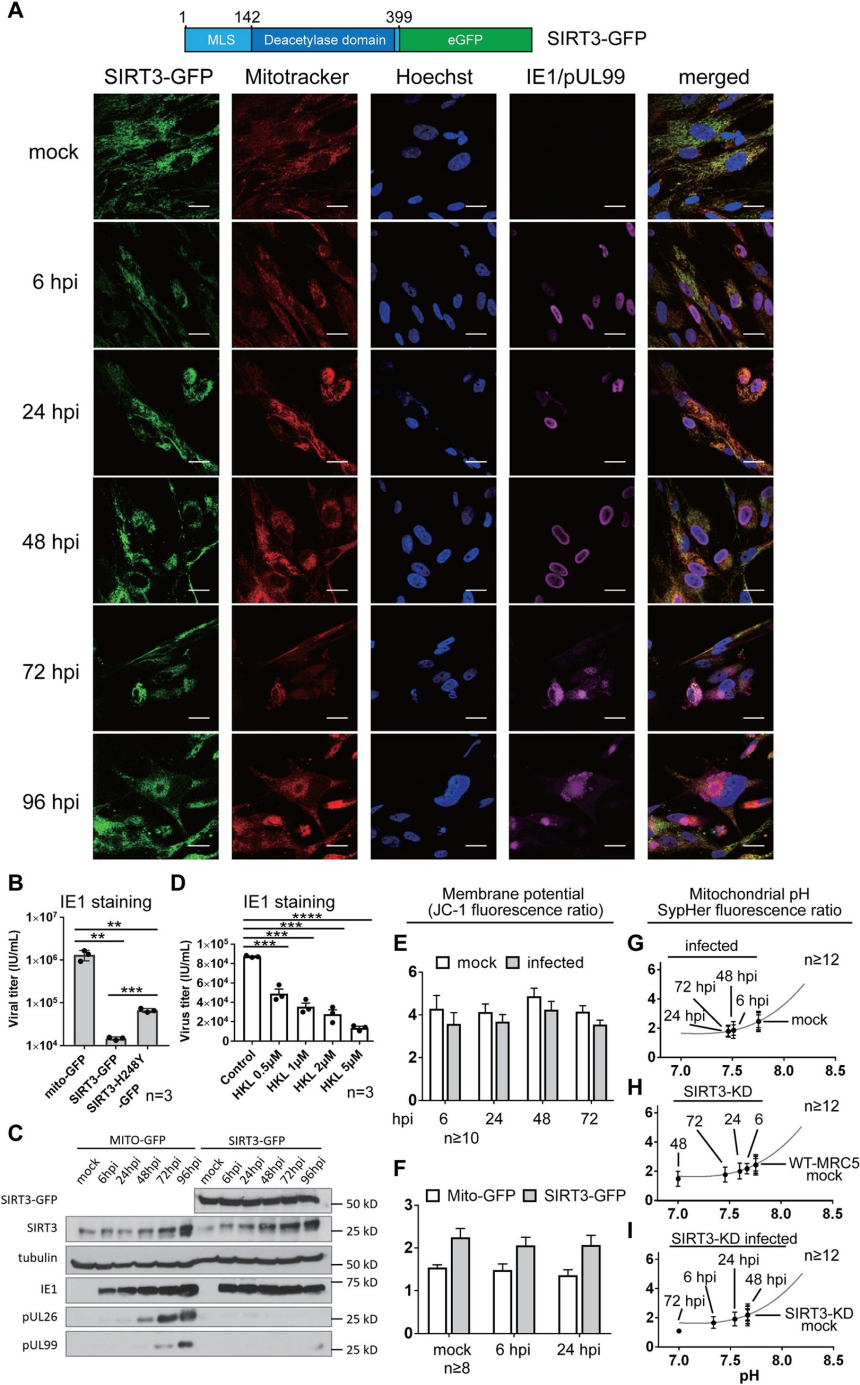
Mitochondrial deacetylase SIRT3 antagonizes HCMV infection and maintains mitochondrial integrity. A. Confocal images of SIRT3-GFP during HCMV infection. SIRT3-GFP cells are stained for IE1 in mock, 6 hpi, 24 hpi, and 48 hpi; pUL99 instead of IE1 was used as the viral marker at 72 hpi and 96 hpi. Cells were treated with MitoTracker Red CMXRos to visualize mitochondria. Schematic of SIRT3-GFP construct is shown above images, where mitochondrial localization (MLS) and the cleavage site are indicated. Scale bar = 10 μm. B. HCMV titer during over-expression of mito-GFP, SIRT3 WT and mutant SIRT3. ** p-value < 0.01, *** p-value < 0.005. Supernatants were collected at 120 hpi for titration by IE1 staining. Three biological replicates were used for each group. C. Viral protein abundances during infection in the context of mito-GFP or SIRT3 over-expression by western-blot. IE1, pUL26, and pUL99 are used as viral markers for immediate early, early, and late stages, respectively. D. Virus titer resulting from the treatment of SIRT3 activator, honokiol (HKL). *** p-value < 0.005, **** p-value < 0.001. Supernatants were collected at 120 hpi and subjected to titration by IE1 staining, with three biological replicates for each group. E. Mitochondrial membrane potential during HCMV infection quantified by JC-1 fluorescence ratio. n > 75 cells for each condition. F. Mitochondrial membrane potential at immediate early and early stages during infection in the context of SIRT3 over-expression. n > 75 cells for each condition. G. Mitochondrial pH during viral infection measured by SypHer fluorescence ratio. n > 75 cells (at least 12 images per condition) for each condition. H. Mitochondrial pH in uninfected cells with SIRT3 siRNA-mediated knockdown. n > 75 cells (at least 12 images per condition) for each condition. I. Mitochondrial pH during infection upon SIRT3 siRNA-mediated knockdown. n > 75 cells (at least 12 images per condition) for each condition.

Although most acetylation sites on mitochondrial proteins still do not have known functions, acetylation has been shown to inhibit several core mitochondrial enzymes [27,41,55–57]. Since HCMV infection induces broad increases in protein acetylation, the removal of these modifications could be critical for restoring mitochondrial health and regulating metabolism. Therefore, we turned our attention to SIRT3, the main mitochondrial deacetylase. We previously reported that siRNA-mediated knockdown of SIRT3 leads to elevated HCMV titer [32]. This suggested that SIRT3 is an antiviral factor, but this role and its possible dependence on deacetylase activity remained to be demonstrated. To investigate SIRT3 function during HCMV infection, we generated fibroblasts stably expressing SIRT3 fused to green fluorescent protein (GFP) (Fig 2A, *top*). SIRT3-GFP localized exclusively to the mitochondria during infection (Fig 2A, *bottom*). The presence of infection was confirmed by monitoring viral proteins (the immediate early protein IE1 at 6, 24, and 48 hpi, and pUL99 at 72 and 96 hpi). Overexpression of SIRT3-GFP resulted in reduced virus titers, measured by both counting of IE1-positive cells in reported plates or by TCID50 (Figs 2B and S2A), supporting the previous report following SIRT3 knockdown [32]. To determine when during infection SIRT3 exerts its antiviral function, we assessed markers of different stages of HCMV replication (Fig 2C). SIRT3 overexpression did not impact the levels of the immediate early protein IE1, while leading to decreased abundances of pUL26 and pUL99, markers of early and late stages of infection. This observation suggests that SIRT3 exerts an antiviral effect starting at an early stage of infection.

To test whether SIRT3 deacetylase activity is necessary for its antiviral function, we constructed a deacetylation-deficient SIRT3 (H248Y) [31] and generated fibroblasts stably expressing this mutant. Compared to the overexpression of wild type (WT) SIRT3-GFP, the mutant partially rescued the inhibition of virus production, as determined by both IE1 staining and TCID50 (Figs 2B and S2A). In line with the titers, the H248Y SIRT3 mutant failed to suppress viral protein levels at the level of wild-type SIRT3 (S2B Fig). To further confirm this, we treated fibroblasts with honokiol (HKL), which has been reported to induce SIRT3 deacetylase activity and levels [58]. SIRT3 activation led to reduced production of virus particles in a HKL concentration-dependent manner (Fig 2D). These results demonstrate that the deacetylase activity of SIRT3 contributes to its antiviral function.

As mitochondrial structure is altered during HCMV infection, it is possible that one facet of SIRT3 antiviral function is its ability to maintain mitochondrial integrity. One hallmark of mitochondrial integrity is the mitochondrial membrane potential (MMP), and both reduced and increased MMP have been reported upon HCMV infection using different biological systems and assays [48,59–61]. Using confocal microscopy and measuring the fluorescence ratio of JC-1, we observed that MMP decreases throughout virus infection in HCMV infected fibroblasts (Fig 2E). We further investigated whether SIRT3 regulates the MMP during infection, and found that stable overexpression of SIRT3-GFP results in increased JC-1 fluorescence ratio early in infection (6 and 24 hpi) when compared to control cells expressing GFP fused to a mitochondrial localization signal (Mito-GFP) (Fig 2F). As the proton gradient accounts for a portion of MMP, we hypothesized that the mitochondrial pH is also affected by infection and modulated by SIRT3. Indeed, in HCMV infected cells, mitochondrial pH was decreased from 7.7 to 7.5 (Fig 2G). SIRT3 siRNA-mediated knockdown (validated in S2C Fig) resulted in decreased mitochondrial pH in uninfected cells (Fig 2H), and this decrease was exacerbated during infection (Fig 2I). These results suggest SIRT3 can help to support mitochondrial integrity during infection, being involved in the maintenance of the MMP and mitochondrial pH.

### SIRT3-substrate interactions are temporally regulated during HCMV infection

**Fig 3:**
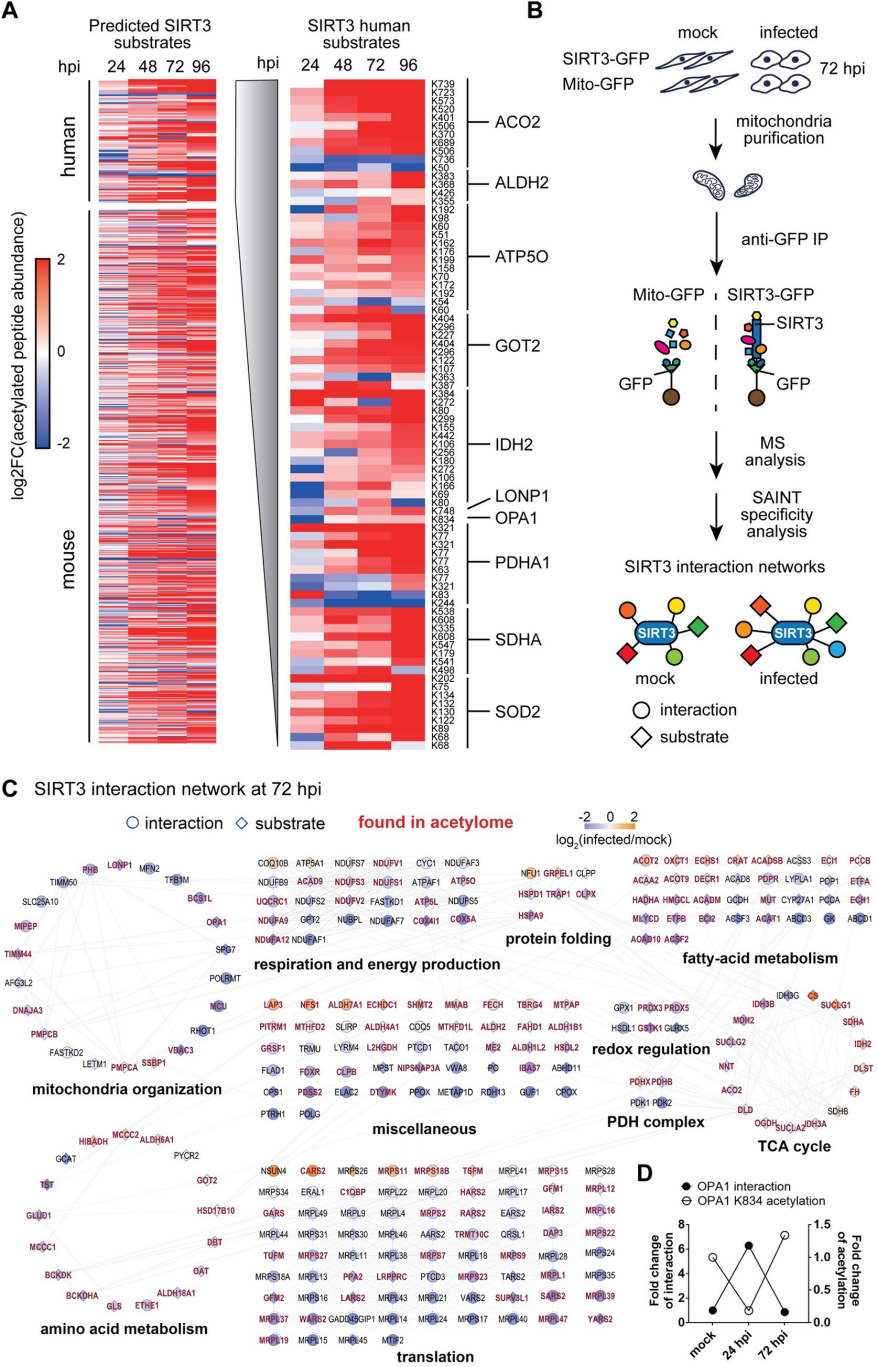
SIRT3 interactions with substrates, including the fusion factor OPA1, are disrupted during HCMV infection. A. Acetylated peptide abundances of known and predicted SIRT3 substrates in human and mouse, relative to mock. Acetylated peptides for each substrate are color-coded by their abundances (log2 fold changes). SIRT3 human substrates are illustrated, and the corresponding acetylation sites and proteins are labeled. B. IP-MS workflow for identifying SIRT3 interactions. C. Interaction network of SIRT3 at 72 hpi. Specific interacting proteins, including those found in the acetylome dataset (red), are grouped by functions, and color coded by their log2 enrichment levels (spectral counts normalized to mock). Circles indicate interaction partners, while diamonds represent putative and characterized SIRT3 substrates. Red font denotes proteins found in the acetylome dataset. D. Fold changes of OPA1 interaction and acetylation levels of lysine 834 at 24 and 72 hpi.

As our results indicate that SIRT3 deacetylase activity contributes to its antiviral function and that SIRT3 regulates the MMP and mitochondrial pH during infection, we investigated our acetylome dataset for the presence of putative SIRT3 substrates (S3 Table). Most knowledge regarding SIRT3 substrates derives from mouse Sirt3 knock-out studies [35], with several substrates also being functionally characterized in human cell studies [37]. We found >130 of these SIRT3 substrates in our infected acetylome dataset (Figs 3A and S3A). Temporal profiles of substrate acetylation were examined with and without normalization to corresponding protein abundances during HCMV infection (Figs 3A and S3B). The infection-induced increase in acetylation was also evident on SIRT3 substrates, with a marked global upregulation by 72 hpi.

These changes in acetylation may represent altered SIRT3-substrate interactions during infection. To address this possibility, we enriched mitochondria by density gradient ultracentrifugation and performed large-scale immunoaffinity purifications (IP) of SIRT3-GFP from uninfected (mock) and infected cells, in parallel to control GFP isolations from Mito-GFP cell lines (Fig 3B). We started by focusing on the 72 hpi time point, when the pronounced global increase in acetylation was evident for many SIRT3 substrates (Fig 3A). Proteins co-isolated with SIRT3 were analyzed by nLC-MS/MS, the interaction specificity was assessed using SAINT [62], and the resulting SIRT3 interaction network was integrated with information from the acetylome analysis (Fig 3C and S4 Table). Demonstrating the ability of this approach to capture enzyme-substrate associations, we identified 137 of its validated or predicted substrates [33–37], as well as 148 proteins that we detected in our acetylome dataset.

Relative quantification of interactions demonstrated that HCMV infection induces a range of alterations in SIRT3 associations with substrates that function in regulating mitochondrial structure and cellular metabolism. In agreement with the overall global increase in the mitochondrial acetylome during infection, many interactions displayed decreased levels (Fig 3C). Among these were SIRT3 associations with proteins involved in mitochondrial organization, including OPA1, a dynamin-related guanosine triphosphatase mutated in dominant optic atrophy. OPA1 is a mitochondrial fusion factor localized at the IMM [63,64] and known to regulate the balance between mitochondrial fusion and fission in response to stress and altered MMP [65]. In uninfected cells, SIRT3-mediated deacetylation of OPA1 K931 was reported to activate this fusion factor [27]. Our acetylome analysis during infection pointed to another OPA1 acetylated residue, K834. This OPA1 acetylation was dynamic during infection, decreasing at 24 hpi, then returning to its original levels, and slightly increasing later in infection (Fig 3A). Our IP-MS study showed that the SIRT3-OPA1 association decreases at 72 hpi compared to mock cells (Fig 3C), in agreement with the slight increase in OPA1 acetylation at that time point. To further verify whether the change in OPA1 acetylation tracks oppositely to its SIRT3 interaction, we performed an additional IP of SIRT3 at 24 hpi, followed by a targeted MS analysis of OPA1. Indeed, we observed increased SIRT3-OPA1 interaction at 24 hpi (Fig 3D and S4 Table). Altogether, our results revealed that SIRT3 displays infection-induced changes in its association with the fusion factor OPA1, which are temporally dynamic and that match K834 acetylation levels on this substrate.

### The SIRT3 substrate OPA1 restricts virus production, a function modulated by its K834 and K931 acetylation status

**Fig 4:**
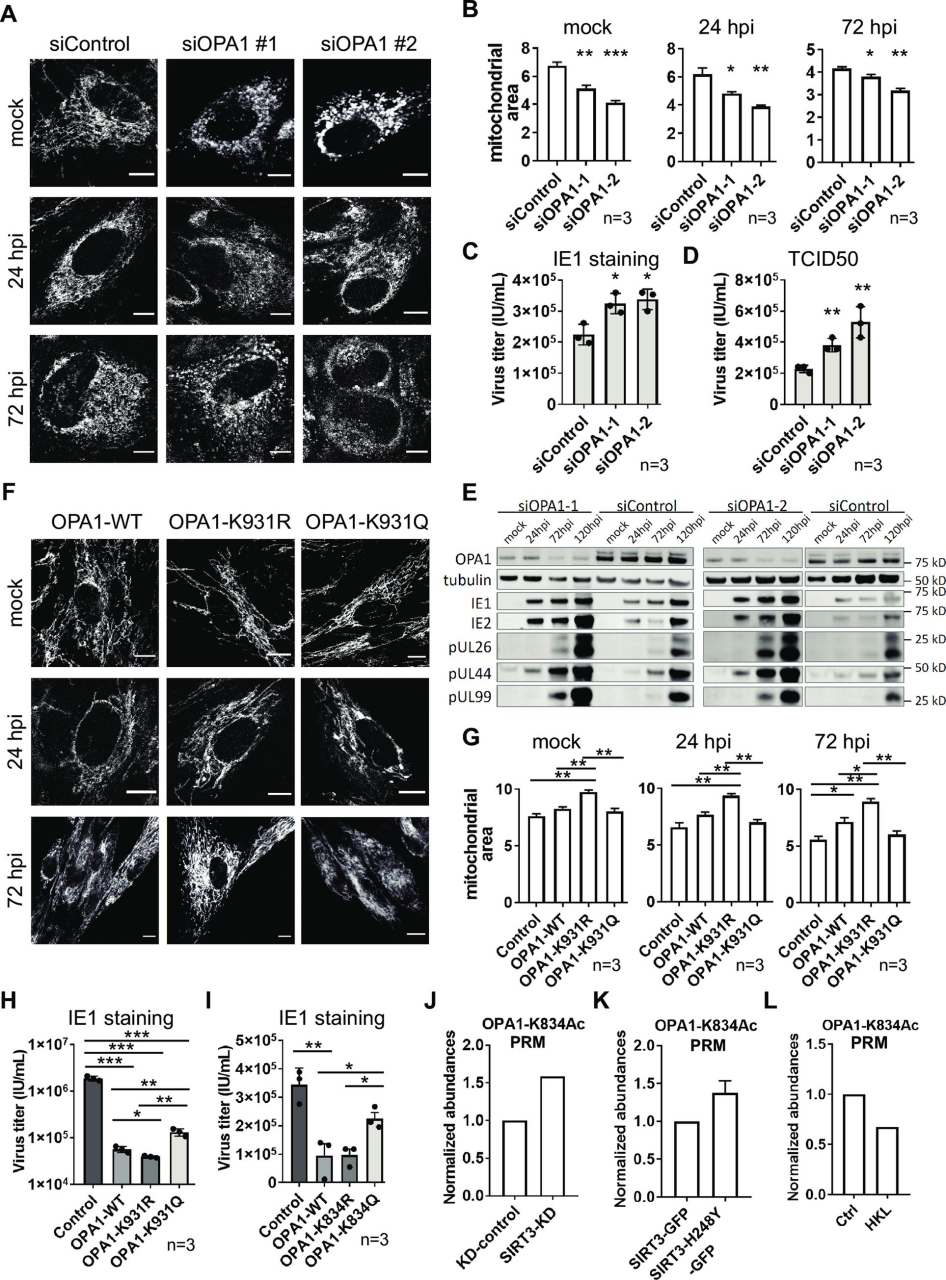
OPA1 acetylation at SIRT3-targeted sites K834 and K931 modulates mitochondrial fission-fusion balance and impacts viral production. A. Representative images of cells with treatment of vehicle or OPA1 siRNA-mediated knockdown (constructs #1&2) at mock, 24 and 72 hpi. MitoTracker Red CMXRos was used as mitochondria marker. Scale bar = 10 μm. B. Mitochondrial area of cells with OPA1 knockdown at mock, 24 and 72 hpi. Mitochondrial area was calculated based on MitoTracker Red CMXRos signal. * p-value < 0.05, ** p-value < 0.01, *** p-value < 0.005. Three biological replicates were used for each group (n > 70 cells per replicate). C-D. HCMV titers of cells with OPA1 knockdown determined by IE1 staining (C) and TCID50 (D). The supernatants were collected in triplicates and subjected to titration at 120 hpi. * p-value < 0.05, ** p-value < 0.01. E. Viral protein abundances at different stages of infection in the context of siOPA1 knockdown. IE1, IE2 were selected as viral marker proteins for immediate early stage; pUL26 and pUL44 for early stage; pUL99 for late stage. F. Representative images of mitochondria (MitoTracker Red CMXRos) in cells expressing OPA1 WT or mutants at mock, 24 and 72 hpi. Scale bar = 10 μm. G. Mitochondrial area quantification in cells expressing OPA1 WT, K931R, or K931Q at 24 hpi and 72 hpi. Mitochondrial area was calculated based on MitoTracker Red CMXRos signal. * p-value < 0.05, ** p-value < 0.01. Three biological replicates were used for each condition (n > 70 cells per replicate). H. HCMV titers of cells over-expressing OPA1 WT or K931 mutants. * p-value < 0.05, ** p-value < 0.01, *** p-value < 0.005. Supernatants were collected at 120 hpi in triplicates and subjected to IE1 staining reporter plate assays. I. HCMV titers of cells over-expressing OPA1 WT or K834 mutants. * p-value < 0.05, ** p-value < 0.01. Supernatants were collected at 120 hpi in triplicates and subjected to IE1 staining reporter plate assays. J-L. PRM quantification of OPA1 K834 acetylation in cells with siSIRT3-KD (J), SIRT3 WT or mutant expression (K), or honokiol treatment (L).

Given our finding of altered OPA1 acetylation status and interaction with SIRT3 during HCMV infection, we examined its function by performing siRNA-mediated knockdown (siOPA1-KD) and over-expression (OE) studies. The decreased or increased OPA1 levels, confirmed by western blot (S4A Fig), did not induce noticeable cell death (Trypan blue staining, S4B Fig). Assessment by live cell microscopy showed that OPA1 knockdown promoted mitochondrial fragmentation (Fig 4A), while increased mitochondrial fusion was evident upon OPA1-OE (Fig 4F) in both uninfected and infected cells. Specifically, OPA1-KD resulted in decreased mitochondrial area (Fig 4B and S5 Table) and increased percentage of mitochondrial fragments (S4C Fig), phenotypes already evident at an early infection time point (24 hpi) and maintained later in infection (72 hpi). Furthermore, OPA1-KD resulted in increased virus titers, as shown by counting IE1-positive cells in a reporter plate or by TCID50 (Fig 4C and 4D). Examination of viral protein abundances in infected cells upon OPA1 knockdown (siOPA1-1 and siOPA1-2) revealed overall elevated levels of immediate early (IE2), early (pUL26 and pUL44), and late (pUL99) viral proteins (Fig 4E, lower exposure in S4D Fig, and quantification in S4E Fig). In contrast, OPA1-OE inhibited virus-induced mitochondrial fragmentation. This is evidenced by the increased mitochondrial area (Fig 4G) and filamentous network and the decreased percentage of fragmented mitochondria (S4G Fig) at both early (24 hpi) and late (72 hpi) stages of infection (S5 Table), in conjunction with decreased HCMV titer (Fig 4H; control versus OPA1 WT). These results suggest that the OPA1-modulated balance between mitochondrial fusion and fission early in infection is critical for virus production.

We next asked whether the OPA1 acetylation status specifically contributes to this observed impact on mitochondrial morphology during infection and virus production. Given the knowledge that, in uninfected cells, SIRT3 has the ability to remove the OPA1 inhibitory acetylation at K931 [27], we first investigated this residue in the context of HCMV infection. We constructed OPA1 mutants with a charge mimic of the unmodified residue (K931R) and a constitutively acetylated mimic (K931Q) (S4F Fig) and assessed their impact on mitochondria structure during infection. Microscopy analyses indicated that fragmented mitochondria were more prevalent in OPA1-K931Q cells compared to those expressing the K931R mutant at both 24 hpi to 72 hpi (Figs 4F and 4G and S4G, and S5 Table). Specifically, mitochondrial area and filamentous appearance were decreased in OPA1-K931Q cells compared to K931R cells (Figs 4G and S4G). Additionally, expression of OPA1-K931R resulted in decreased virus titer at a similar extent as OPA1-WT, while the expression of OPA1-K931Q mutant led to a partial rescue of the virus titer (Fig 4H). These results indicate that the presence of the acetyl-mimic at K931, which inhibits OPA1 function, allows for mitochondrial fragmentation to proceed and is beneficial for virus production.

As our acetylome and interactome studies highlighted the dynamic nature of OPA1 K834 acetylation, a site not previously investigated, we next sought to determine whether this residue impacts HCMV production similarly to K931. By generating K834Q and K834R mutants, and expressing these in human fibroblasts, we found that virus titers are higher in cells expressing the acetyl-mimic mutant (K834Q) when compared to the WT and the charge-mimic OPA1 mutant (K834R, Fig 4I). Therefore, the acetyl-mimic mutant at K834Q also inhibited OPA1, aiding virus production. As these results point to K834 as a previously unrecognized OPA1 regulatory hub, we tested whether this residue is a substrate of SIRT3. Antibodies against this acetylated site are not available, so we designed a targeted MS assay based on parallel reaction monitoring (PRM) for the site-specific detection and quantification of OPA1 K834 acetylation. Indeed, SIRT3-KD led to increased K834 acetylation levels (Fig 4J), and cells expressing the deacetylation-deficient SIRT3 mutant (H248Y) had higher levels of OPA1 K834 acetylation than those expressing the WT SIRT3 (Fig 4K). Additionally, SIRT3 activation using HKL resulted in reduced K834 acetylation levels (Fig 4L), supporting the dependence on SIRT3 enzymatic activity for deacetylating this residue. Altogether, these results establish K834 acetylation as a previously unrecognized target for SIRT3 activity and indicate that OPA1-mediated fusion restricts virus production, a function partly controlled via its acetylation status at K834 and K931.

### SIRT3 deacetylase activity functions to balance mitochondrial fragmentation and fusion

**Fig 5:**
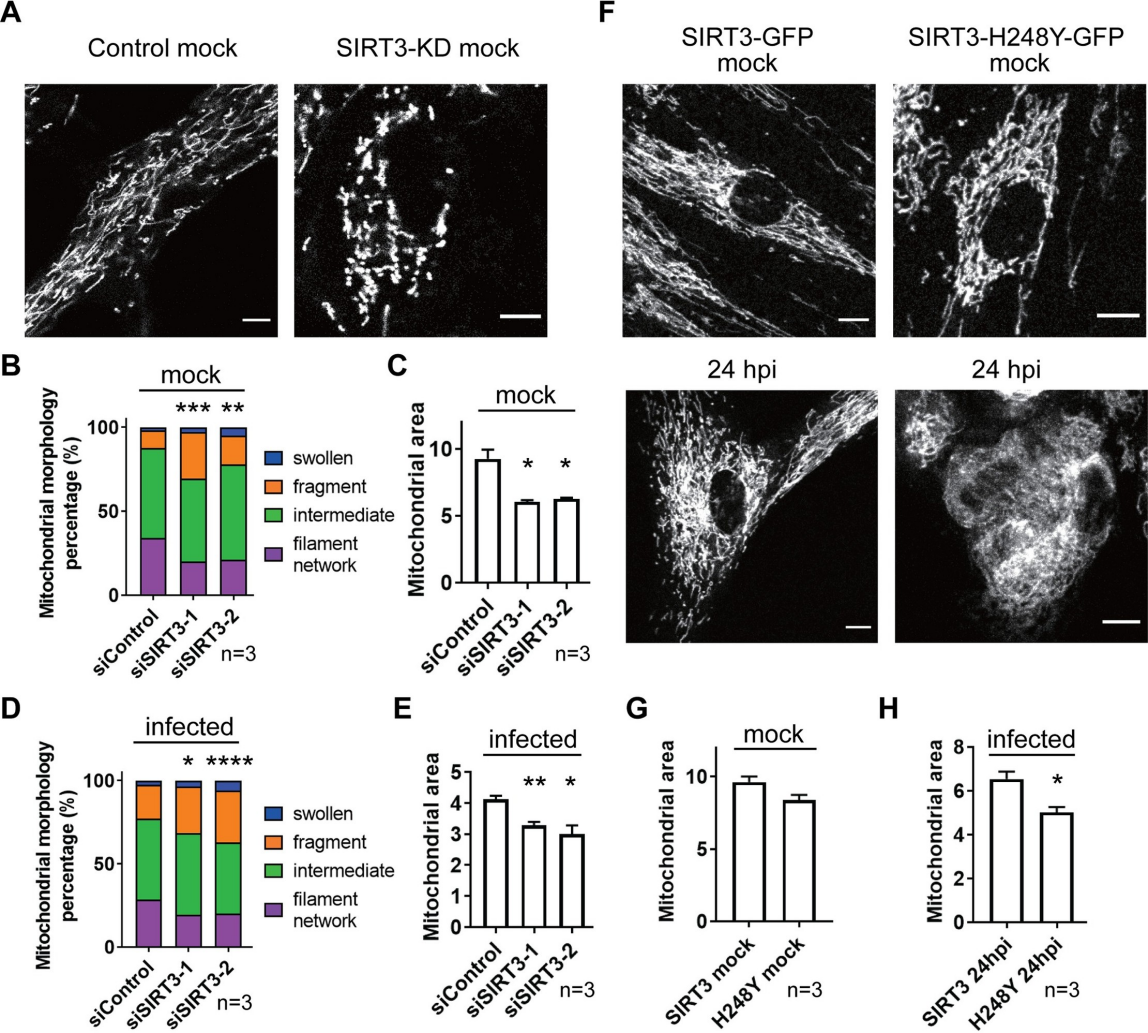
Control of SIRT3 levels or deacetylase activity is sufficient for modulating mitochondrial fusion. A. Representative microscopy images of mitochondria (visualized by MitoTracker Red CMXRos) in the context of SIRT3 siRNA-mediated knockdown (#1). Scale bar = 10 μm. B-C. Mitochondrial morphology and mitochondrial area of uninfected cells with SIRT3 siRNA knockdown. MitoTracker Red CMXRos was used to stain the mitochondria. Student t-test was conducted to determine the statistical differences in fragment between control and knockdown groups. * p-value < 0.05, ** p-value < 0.01, *** p-value < 0.005. Three biological replicates were used for each group (n > 60 cells per replicate). D-E. Mitochondrial morphology and mitochondrial area of infected cells (72 hpi) treated with SIRT3 siRNA constructs. Mitochondria were stained with MitoTracker Red CMXRos. Student t-test was conducted to determine the statistical differences in fragment between control and knockdown groups. * p-value < 0.05, ** p-value < 0.01, **** p-value < 0.001. Three biological replicates were used for each group (n > 60 cells per replicate). F. Representative microscopy images of mitochondria (MitoTracker Red CMXRos) in uninfected (upper) and infected (24 hpi, lower) cells expressing of SIRT3 WT or deacetylation-deficient mutant. Scale bar = 10 μm. G-H. Mitochondrial area quantification (based on MitoTracker Red CMXRos signal) of uninfected (G) and infected (H) cells expressing either SIRT3 mutant or WT. * p-value < 0.05. Three biological replicates were used for each group (n > 60 cells per replicate).

Our finding that SIRT3 displays reduced interactions with its substrate OPA1 late in infection, and that active SIRT3 and OPA1 both inhibit virus production, led us to ask whether the SIRT3 deacetylase activity by itself has the ability to impact mitochondrial structure. This would also be supported by the SIRT3 interactions with other proteins involved in mitochondrial organization (Fig 3C). More broadly, the large number of mitochondrial SIRT3 substrates and their overall increase in acetylation levels during infection suggest a link between the global mitochondrion acetylation status and its structure. Indeed, we find that modulation of SIRT3 levels is sufficient for regulating mitochondria structure. SIRT3 knockdown led to increased mitochondrial fission (Fig 5A), in contrast to the reticular mitochondria in the control cells. Quantification of the different types of mitochondrial structures showed decreased filamentous mitochondria (Fig 5B) and overall mitochondrial areas (Fig 5C), in conjunction with increased percentages of fragmented mitochondria in uninfected cells (Fig 5B and S5 Table). These changes in mitochondrial structure were recapitulated in infected cells, illustrated for 72 hpi (Fig 5D and 5E).

To determine the contribution of the SIRT3 enzymatic activity to this regulation of mitochondrial morphology, we investigated cell lines stably expressing SIRT3 WT or the deacetylation-deficient mutant H248Y. Microscopy examination and quantification of relative mitochondrial areas showed that the cells expressing the H248Y mutant showed more pronounced fragmentation and reduced areas compared to the cells expressing WT SIRT3 in both uninfected and infected cells (Fig 5F–5H). Altogether, these data support a role for SIRT3 and its deacetylase activity in regulating mitochondrial morphology.

### HCMV infection disrupts the interaction of the antiviral protein ACAA2 with SIRT3, promoting its acetylation

In addition to substrates involved in mitochondrial organization, our interactome study demonstrated that SIRT3 also had infection-induced changes in interactions with substrates involved the TCA cycle, fatty-acid metabolism, and amino acid metabolism (Fig 3C). Previous metabolomic studies have shown that HCMV induces major changes to the cellular metabolism [66,67]. Therefore, it is possible that SIRT3 deacetylase activity functions in host defense not only by maintaining mitochondrial structural integrity, but also by regulating cellular metabolism.

**Fig 6:**
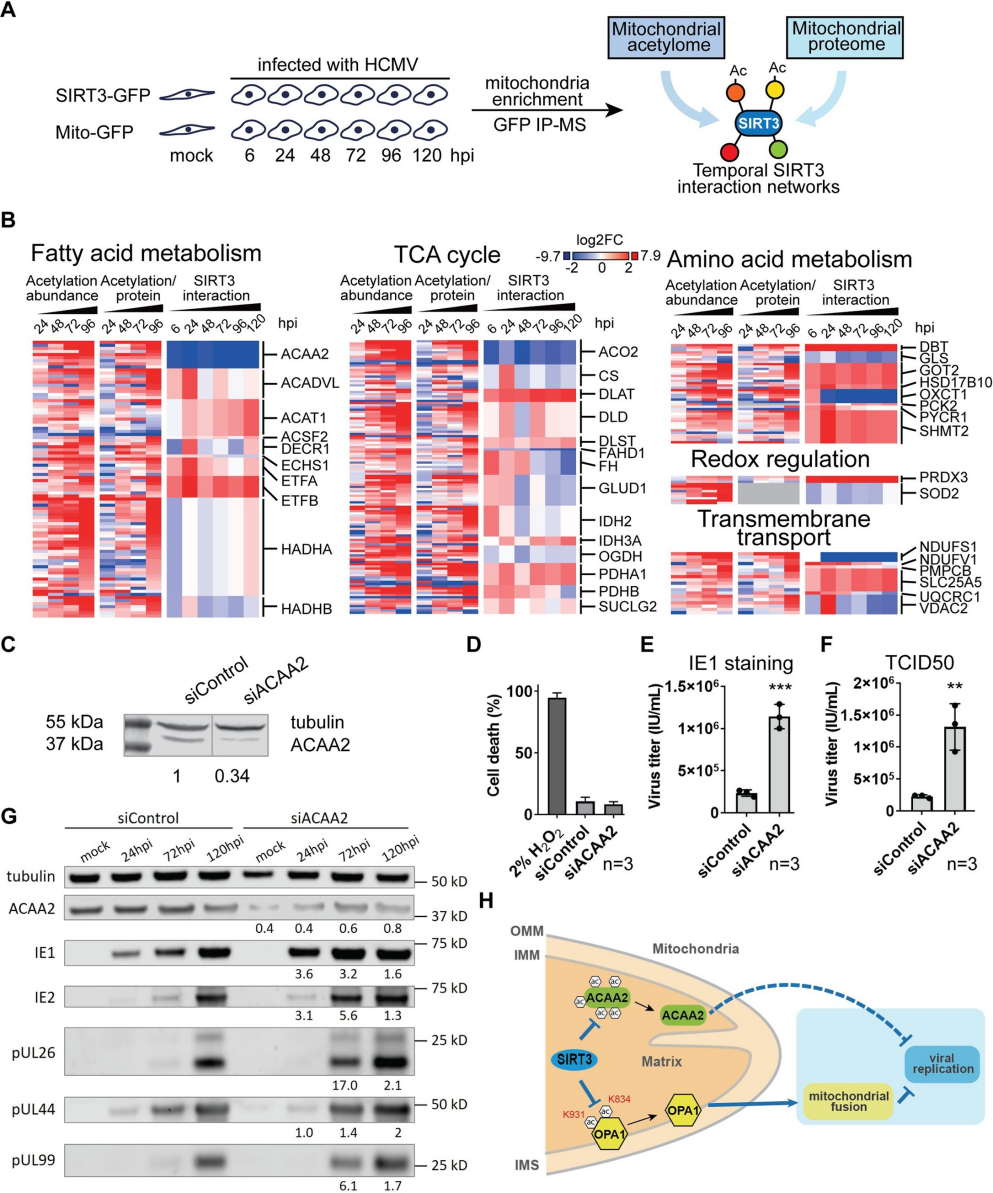
ACAA2 is an HCMV restriction factor, inhibited during infection via loss of SIRT3 interaction and increased acetylation. A. Workflow for determining temporal SIRT3 interactions with mitochondrial proteins during infection using small-scale IPs. B. Integration of acetylation abundances, acetylation levels normalized to protein abundances, and SIRT3 interactions during the course of infection. Representative SIRT3 interacting proteins are presented and grouped by key mitochondrial metabolism pathways, i.e. fatty acid metabolism, TCA cycle, amino acid metabolism, redox regulation and transmembrane transport. The abundances of acetylated peptides (left), acetylated peptides normalized to protein abundances (right), and SIRT3 interaction levels (right) are grouped by the proteins. Colors denote the log2 fold change relative to mock. C. Validation of ACAA2 siRNA-mediated knockdown by western blot. Values below the blot indicate the ACAA2 densitometry ratios normalized to tubulin. D. Cell viability upon ACAA2 KD. Cell death percentages were measured using Trypan blue with hydrogen peroxide as the positive control. n = 3 technical replicates for each group. E-F. Viral titers upon ACAA2 KD measured by IE1 staining reporter plate (E) and TCID50 (F). The supernatants were collected in triplicates and subjected to titration assays at 120 hpi. Student t-test was conducted. ** p-value < 0.01, *** p-value < 0.005. G. Viral protein abundances during infection in the context of siACAA2 knockdown. IE1 and IE2 are used as viral marker proteins for immediate early stage; pUL26 and pUL44 for early stage; pUL99 for late stage. H. Model for SIRT3 regulation of OPA1 and ACAA2 during viral infection. SIRT3 activates OPA1 by removing acetylation on lysines 834 and 931, which upregulates mitochondrial fusion and subsequently interferes with viral production. SIRT3 associates with ACAA2, reducing its acetylation modifications, likely promoting fatty acid oxidation. ACAA2’s function antagonizes viral replication. The function of ACAA2 has been reported to be involved in multiple steps during viral infection, regulating transcription, apoptosis and fatty acid metabolism, but the exact mechanism underlying its antiviral effect remains to be investigated.

Metabolic changes have been reported to occur at earlier infection time points than our 72 hpi SIRT3 interaction study, with increased flux through glycolysis and elevated fatty acid synthesis observed already by 48 hpi [20,66]. To gain a better temporal resolution of SIRT3 interactions with metabolic factors, we performed small-scale IPs of SIRT3 at time points covering the entire HCMV replication cycle—mock, 6, 24, 48, 72, 96, and 120 hpi in biological triplicate (Fig 6A). Overlay of this temporal interactome with our acetylome and proteome datasets enabled us to search for opposing changes in interaction and acetylation, which would suggest the involvement of the SIRT3 deacetylase function. Evident temporally-regulated interactions were with proteins involved in fatty acid metabolism, TCA cycle, amino acid metabolism, redox regulation, and transmembrane transport (Fig 6B and S6 Table).

The most pronounced interaction change, observed immediately upon infection, was the loss of SIRT3 association (by > 3 fold, not detected at 6, 24, 48, 96, and 120 hpi) with the acetyl-CoA acyltransferase 2 (ACAA2, also known as 3-Ketoacyl-CoA thiolase), a predicted substrate of SIRT3 (Fig 6B) [33,34,40]. This decreased interaction with SIRT3 aligned with an increase in ACAA2 acetylation levels during infection. ACAA2 is the thiolase that catalyzes the last step of the mitochondrial beta-oxidation pathway. In addition to its functions in fatty acid metabolism, ACAA2 was described to have roles in regulating cellular apoptosis and transcription [68,69]. As HCMV relies on elevated fatty acid synthesis for its virus envelopment [20], as well as on inhibition of cellular apoptosis [46,48,70] and regulation of cellular transcription [71–73] for its virus replication, ACAA2 may have the ability to affect HCMV production via different mechanisms. To test its function, we knocked down ACAA2 in MRC5 cells using siRNA and confirmed knockdown efficacy (Fig 6C) and lack of induced cell death (Fig 6D). ACAA2 knockdown resulted in significantly increased virus titers, when measured by either counting IE1-positive cells in reporter plates or TCID50 (Fig 6E and 6F). In agreement with our virus titer results, ACAA2 KD also resulted in increased levels of viral proteins. The impact of the ACAA2 KD was observed at 24 hpi and maintained throughout the HCMV replication (Fig 6G), suggesting a role for ACAA2 early in infection. The observed increase in viral protein levels and viral titers upon ACAA2 knockdown suggest an antiviral role for ACAA2.

Altogether, these results demonstrate that protein acetylation underlies mechanisms regulating both mitochondrial structure and metabolism. The specific acetylated proteins that we investigated provided examples of how the HCMV-induced increase in the mitochondrial acetylome provides an effective mean to inhibit enzymes involved in the mitochondrial fusion/fission balance and fatty acid oxidation. Therefore, SIRT3 promises to offer a single target that, when activated, can restore mitochondrial integrity and health. This is supported by our observation that SIRT3 deacetylase activity contributes to its antiviral function, and that the regulation of SIRT3 levels is sufficient for modulating mitochondrial structure.

## Discussion

Alterations in mitochondrial structure and functions are at the core of numerous types of viral infections, representing mechanisms through which viruses inhibit host immune responses and apoptosis, and regulate cellular metabolism. Early in HCMV infection, calcium influx into the mitochondria disrupts mitochondrial integrity and causes fragmentation [17]. Additionally, mitochondrial pathways are rewired during infection to support virus production. For example, apoptosis is inhibited, while the TCA cycle fluxes and fatty acid synthesis are induced [20]. These disruptions in the mitochondrion structure and function are linked to changes in its composition, including an overall upregulation of mitochondrial protein abundances and altered metabolite levels [19–21,74]. Recently, we observed that the mitochondrial acetylome is also upregulated during HCMV infection [29]. Considering that acetyl-CoA levels are increased by ~8 fold in infected cells [53], our finding suggests that infection may induce global, non-enzymatic acetylation, a possibility supported by our temporal acetylation and motif assessment in the current study. Certain aspects of this increased acetylation may be beneficial for HCMV replication, as we previously reported an antiviral role for the mitochondrial deacetylase SIRT3 [32]. The underlying mechanism of this SIRT3 function or its dependence on its enzymatic activity remained unknown. SIRT3 is the main lysine deacetylase in the mitochondria, and several of its substrates have been functionally characterized in uninfected cells, including PDHA1 [75], LCAD [41], and SOD2 [76]. However, no study has thus far attempted to characterize SIRT3 substrates and the function of their site-specific acetylations during viral infection. Given the breath of SIRT3 mitochondrial substrates, we predicted that SIRT3 exerts its antiviral effect by catalyzing deacetylation of its substrates, which in turn impacts various facets of mitochondrial functions relevant to virus production. In this study, we aimed to decode the dynamics of SIRT3-substrate interactions and the role of the resulting temporal acetylation events on mitochondrial structure, metabolism, and production of virus progeny upon HCMV infection.

We establish that the SIRT3 deacetylase activity contributes to its antiviral function, suggesting that the regulation of the mitochondrial acetylation status impacts the progression through the virus replication cycle and virus production. We next asked whether SIRT3 activity can modulate mitochondrial integrity during infection. The mitochondrion morphology is under a finely-tuned control during HCMV infection. The HCMV protein pUL37x1 promotes mitochondrial fragmentation to prevent apoptosis [14,17], whereas pUS9 targets the mitochondria to induce leakage of and inhibit the antiviral factor MAVS [77,78]. Given that among the confirmed and predicted SIRT3 substrates are proteins involved in regulating mitochondrial structure, it is possible that an active SIRT3 could tilt the balance between mitochondrial fusion and fission, thus interfering with the virus replication cycle. Indeed, using microscopy, we demonstrate a role for the SIRT3 deacetylase activity in promoting mitochondria filamentous structure. Our results suggest the mitochondrial acetylation status is broadly linked to the control of mitochondrial structure, adding to the emerging knowledge from uninfected cells that lysine acetylation on fission and fusion proteins can provide a toggle for mitochondrial structure. For example, high fat diet leads to increased acetylation on K642 and activation of the mitochondrial fission protein dynamin-related protein 1 (DRP1) [79]. The acetylation on OPA1 at K931 was reported to suppress its pro-fusion function, and SIRT3 activates OPA1 by deacetylating this site [27]. We discover that OPA1 knockdown leads to mitochondrial fission during infection and elevated virus titers, while OPA1 overexpression resulted in reduced virus titers. Mutation of OPA1 to a K931 acetylation mimic compromised its pro-fusion ability and alleviated the inhibition of viral titer by OPA1 overexpression. Additionally, we uncover OPA1 K834 as an acetylated residue that also inhibits the ability of OPA1 to suppress virus production and is a target of SIRT3 deacetylase activity. These findings support a link between mitochondrial structure and viral production, where mitochondrial fission aids production of infectious viral particles, while OPA1-mediated fusion counteracts it (Fig 6H). Furthermore, we find that the SIRT3-OPA1 interaction is temporally regulated during infection, being elevated at an early stage (24 hpi) and then disrupted later in infection (72 hpi). It is possible that this dynamic regulation represents first a host defense response, followed by a virus inhibition of SIRT3 function. We find that OPA1 knockdown and over-expression have an impact on mitochondrial morphology and HCMV viral protein levels at 24 hpi, suggesting that the SIRT3 substrate OPA1 exerts a virus restriction function early in infection. This is in agreement with our observations that SIRT3 acts in antiviral response early in infection and that its deacetylase activity contributes to this function. Altogether, our results from the mutagenesis mitochondrial structure, interaction, and virus titer assays highlight protein acetylation as a critical toggle of mitochondrial structure during HCMV infection.

The transmembrane potential, such as that across the inner mitochondrial membrane, is another regulatory element of mitochondrial integrity. Using a fluorescence microscopy assay, we observed a decrease in MMP during HCMV infection in fibroblasts, in agreement with previous reports [48,59,60]. This is complementary to a previous discovery that mitochondrial biogenesis and respiration rate are induced by HCMV infection [61]. The MMP is directly linked to apoptosis [80], ATP synthesis, transmembrane transport of metabolites and ions, and reactive oxygen species production [81], which, in turn, affect a range of metabolic pathways. Similarly, cellular stress and dysregulation of ions can drive alterations in MMP. The fact that SIRT3 maintains the MMP and pH during infection also guides us to ask how SIRT3 modulates mitochondrial metabolism during infection. To this end, we started by determining the changes in SIRT3 interactions at a late stage of infection, when mitochondrial integrity is already disrupted and changes in cellular metabolism were reported as prevalent [53]. The breadth of the identified interactions and their dynamic nature underscores the versatility of SIRT3 in regulating multiple mitochondrial metabolic pathways during infection. Of note, we found SIRT3 to lose associations with the majority of proteins involved in mitochondrial organization. Our finding agrees with an observation in uninfected cells that, when the membrane potential is disrupted, SIRT3 dissociates from the ATP synthetase on the inner mitochondrial membrane and gains association with matrix proteins [38]. The enhanced interactions between SIRT3 and mitochondrial matrix proteins, such as those involved in the TCA cycle (e.g., SDHA, IDH2, DLST, and FH), may reflect the need for reprogrammed mitochondrial metabolism for virus production. It remains to be determined how these interactions specifically modulate metabolic flux and whether they impact viral production. As HCMV is known to upregulate long-chain fatty acid synthesis for the production of the virion envelope [53], it is worth noting that the SIRT3 interactions with proteins regulating fatty acid metabolism are distinctly regulated. We further explored the temporal dynamics of these SIRT3 interactions by performing IPs throughout the virus replication cycle. The integration of these temporal SIRT3 interactions with measurements of protein abundance, acetylation, and viral titers led us to propose a model (Fig 6H) where HCMV infection disturbs the SIRT3-ACAA2 association, resulting in hyper-acetylation and inhibition of ACAA2, an inhibition that functions to support viral replication. Future studies will be needed to delineate whether this impact on virus replication may derive from the ACAA2 functions in fatty acid metabolism or from its regulatory roles in apoptosis and transcription. It is tempting to speculate that reduced ACAA2 activity, via either its knockdown or acetylation, would lead to the accumulation of beta-ketoacyl-CoA intermediates in the mitochondria, and ACAA2 inhibition (using treatment with 2-bromooctanoate) has been reported to inhibit fatty acid oxidation [82,83]. However, it remains to be seen whether and how ACAA2 acetylation alters the levels of intermediates of fatty acid metabolism. It is also possible that the effects of the ACAA2 knockdown and ACAA2 acetylation are driven via non-metabolic mechanisms, given that ACAA2 was reported to associate with H2A.Z-occupied transcription start sites and enhancers [69] and to regulate apoptosis by interacting with BNIP3 [68]. Therefore, ACAA2 may have roles in regulating viral or host gene expression during infection, as well as apoptosis. Furthermore, although ACAA2 was reported as a likely SIRT3 substrate [33,34,40], it remains to be demonstrated whether ACAA2 is also a target of SIRT3 enzymatic activity during infection and which acetylation sites are regulated by SIRT3. Given that we detected multiple dynamic acetylation sites on ACAA2, are all these sites regulated by SIRT3 or only a subset? Additionally, which specific site modulates ACAA2 activity? Our findings expand the growing knowledge of how enzymes involved in fatty acid metabolism are regulated during infection. These regulatory processes may be relevant during infection with other enveloped viruses that also rely on the increase of fatty acid synthesis, such as other herpesviruses, vaccinia virus, influenza A, hepatitis C virus, and coronaviruses, among other [84,85].

In summary, our study highlights the SIRT3 deacetylase as a bridge that connects protein acetylation status to the functions of proteins regulating mitochondrial integrity and metabolism during HCMV infection. Our findings of altered SIRT3-substrate interactions and global increase in the mitochondrial acetylome can provide a basis for better understanding HCMV-induced pathologies. Excess mitochondrial acetylation and lack of SIRT3 function have both been shown to be detrimental to mitochondrial metabolism [41,86]. Additionally, the dysregulation of mitochondrial metabolism has been implicated in diseases linked to HCMV infection, including the metabolic syndrome [87] and cardiac hypertrophy [88]. We expect our results to also have implications in other biological contexts besides viral infection. Hyper-acetylation of certain mitochondrial proteins is evident in multiple human diseases, such as fatty liver disease [89], cardiovascular defect [86,90], and cancer [91]. Therefore, this work is anticipated to expand our understanding of how global mitochondrial acetylation reflects mitochondrial health or the development of different human diseases.

## Materials and methods

### Cell culture

MRC5 human lung fibroblasts (ATCC) were cultured with 5% CO_2_ at 37°C in Dulbecco’s modified Eagle’s medium (DMEM, Life Technologies) supplemented with 10% (v/v) FBS (Invitrogen) and 1% (v/v) penicillin–streptomycin solution (GIBCO). Test for mycoplasma contamination was conducted, and MRC5 cells were used within ten passages.

### Generation of stable cell lines

C-terminal EGFP-tagged SIRT3 construct was generated by overlap-extension PCR and subcloned into pLXSN-EGFP retroviral vector between the XhoI and BamHI restriction sites. Phoenix-AMPHO (ATCC) cells on a 6 cm dish, at 60% confluence, were transfected with 1 μg of the construct and Lipofectamine 2000 as per protocol (Thermo Fisher Scientific) for 12 hours. The retrovirus-containing supernatant was collected at 48 and 72 hours after transfection. The filtered supernatant was then incubated with fresh MRC5 cells on a 10 cm dish with 400 μg/mL G418 (Sigma-Aldrich) for selection. A control cell line (Mito-GFP) was generated using mitochondrial localization signal (MLS) derived from COX8 in place of SIRT3-GFP.

### Viral growth and infection

HCMV strain AD169 was used as the wild-type strain. For infection, the viral stock was diluted in culture medium and incubated on top of cells at multiplicity of infection (MOI) of 3 at 37°C for 1 hr. The media was then replaced with fresh culture medium, and the infected cells were incubated for the indicated length of time.

### Mitochondrial isolation

MRC5 cells were trypsinized and pelleted at 600 × g for 5 min, followed by 2 × washes in 5 mL ice cold DPBS and pelleting. 6 × 15 cm dishes of cells were used per condition per cell line for large scale SIRT3 IPs, and 3 × 15 cm dishes of cells were used per time point for mitochondrial whole proteome. Mitochondria were enriched using a density-gradient dependent method as previously described. Briefly, cells were incubated in sucrose hypotonic solution B (0.25 M sucrose, 6 mM EDTA, 120 mM HEPES-NaOH pH7.4) for 15 min on ice. Cells were forced through 14 μm polycarbonate membranes (Sterlitech Corporation) twice using a syringe. After pelleting cell lysates at 1400 × g for 10 min at 4°C, the supernatant was pelleted by a second centrifugation at 20,000 × g for 30 min at 4°C. Organelles were resuspended in solution B and loaded on top of a discontinuous gradient of Optiprep (Axis-shield) (0, 10, 15, 20, 25, and 30% (v/v)) mixed with solution B. The gradient was subject to ultracentrifugation in a SW60 Beckman rotor at 120,000 × g for 3 hrs at 4°C. Fractions #3 and #4 were combined and centrifuged to pellet twice in cold PBS at 20,000 × g for 10 min at 4°C. The pelleted mitochondria were stored at -80°C for subsequent MS analyses.

### Determination of SIRT3 interactions at 72 hpi using immunoaffinity purification

SIRT3-GFP and Mito-GFP cells in 6 × 15 cm dishes were infected with HCMV AD169 at a MOI of 3, and the mitochondria pellets were collected at 72 hpi. The enriched mitochondria were gently mixed with 500 μL 20mM HEPES-KOH, pH 7.5, containing 1:100 (v/v) protease inhibitor mixture (Sigma), then flash frozen in liquid nitrogen as droplets, and then subject to cryogenic grinding using a Retsch MM 301 Mixer Mill (Retsch) (10 steps × 2 min at 30 Hz). The grinded mitochondrial powder was suspended in 5 mL of lysis buffer (20 mM HEPES-KOH pH 7.4, 110 mM potassium acetate, 2 mM MgCl_2_, 0.1% Tween-20, 1 M ZnCl_2_, 0.2% Triton X-100, and 1 μM CaCl_2_) containing 1:100 (v/v) protease and phosphatase inhibitor cocktail (Thermo Fisher Scientific). The resulting mitochondria suspension was then homogenized using a Polytron (2 × 15 s) (Kinematica), and centrifuged at 8,000 × g for 10 min at 4°C. The supernatant were incubated with 7 mg of Dynabeads M-270 Epoxy beads (Invitrogen #14301) conjugated with home-made rabbit anti-GFP antibody for 1 hr at 4°C. The beads were washed 4 × with lysis buffer (without protease inhibitors) and twice with distilled H_2_O. The washed beads were then incubated at 70°C with 40 μL of 1 × LDS sample buffer (Invitrogen) for 20 min, and eluted by shaking on a Tomy shaker for 10 min at room temperature. The eluents were reduced and alkylated by 50 mM DTT and 50mM chloroacetamide at 70°C for 20 min.

The IP eluents were partially resolved by 4–12% Bis-Tris NuPAGE gels (Invitrogen) and stained with SimplyBlue Coomassie stain (Invitrogen) for visualization. Sample in each lane on the gel was excised into 1 mm slices, and pooled into six fractions. The gel pieces were then transferred to a 96-well plate and destained with vortex for 15 min in 50 mM ammonium bicarbonate (ABC) and 50% ACN. The samples were then subject to two rounds of dehydration and rehydration using 100% ACN and 50 mM ABC, respectively. The gel pieces were dehydrated in 100% ACN for 15 min and incubated with 12.5 ng/μL trypsin (Promega) in 25 mM ABC. The plate was then properly sealed and incubated with gentle shaking at 37°C overnight for digestion. The peptides were then extracted in 0.5% formic acid and 60% ACN for 15 min at room temperature, followed by two more extractions. The extracted peptides were combined for each fraction, and concentrated by speed-vac.

### MS analysis for SIRT3 interactions at 72 hpi

The resulting peptides were resuspended in 1% TFA, 2% ACN, and analyzed by nLC-MS/MS on a Dionex Ultimate 3000 RSLC coupled to an LTQ-Orbitrap Velos ETD mass spectrometer (ThermoFisher Scientific). The peptides were loaded onto a trap column (Magic C18 AQ, 3 μm, 100 μm × 2.5 cm; Michrom Bioresources) and desalted for 5 min at 5 μL/min with 0.5% TFA, 1% ACN, 98.5% H_2_O. The peptides were then separated by reverse phase chromatography (Acclaim PepMap RSLC, 1.8 μm, 75 μm × 25 cm) using a gradient of 4% B to 16% B over 60 min and 16% B to 40% B over 30 min at a flow rate of 250 nL/min (A: 0.1% formic acid, 0.1% acetic acid in water; B: 0.1% formic acid, 0.1% acetic acid in 97% ACN).

For each fraction sample, data-dependent acquisition was used with FT preview scan disabled and dynamic exclusion of 60 s. MS1 full scans were performed by Orbitrap from 350 to 1700 *m/z* with a resolution of 30,000 at *m/z* = 400. The top 20 precursors were fragmented by CID with the following parameters: isolation width of 2.0 Th, normalized collision energy of 30, and activation time of 10 ms. Targeted values of predictive automatic gain control for Fourier transform full-scan MS and ion trap MS^2^ were set to 1E6 and 1E4, respectively; injection times for FT MS and IT MS^2^ were 500 and 100 ms, respectively. Acquired MS/MS spectra from each fraction sample were searched by Proteome Discoverer (PD ver. 1.3; ThermoFisher Scientific)/SEQUEST (ver. 1.20) against the tryptic reverse and forward human and herpesvirus sequences (downloaded in August 2013) from UniProt-SwissProt appended with common contaminant sequences (21,569 entries).

The following searching parameters were adopted: full tryptic specificity, maximum missed cleavages of 2, precursor and fragment ion mass tolerances of 10 ppm and 0.5 Da, respectively, carbamidomethylcysteine (+57 Da) as a static modification, methionine oxidation, phosphorylation of serine, threonine, and tyrosine, and acetyl-lysine as variable modifications. Peptide spectrum matches were analyzed and assembled into protein groups by Scaffold (ver. 3.6.5; Proteome Software, Inc.) using PeptideProphet and ProteinProphet followed by X!Tandem (GPM 2010.12.1.1) re-search of the peptide spectrum matches with the subset database parameter and additional variable modification of deamidation of asparagine and glutamine (+1 Da), acetylation of lysine (+42 Da), and pyro-Glu formation of peptide N-terminal glutamate (-17 Da). Peptide spectrum matches across all conditions were combined in a single Scaffold session, and the false discovery rate of protein and peptide were set to <1%, and a minimum of two unique peptides per protein in at least one condition is required for protein groups.

### Interaction specificity analysis using SAINT and data normalization

To analyze the specificity of proteins, spectral counts matrix was submitted to Significance Analysis of INTeractome (SAINT) [62] to assess specificity scores. All interactions with an average SAINT probability of > 0.8 in any condition and mitochondrial proteins annotated by MitoCarta 2.0 were accepted as putative specific interactions for further analysis. Spectral counts were normalized to SIRT3 in each biological replicate, and the normalized spectral counts divided by the protein abundances from the mitochondrial proteome dataset were imported to Cytoscape (ver. 3.7.0) to generate the interaction network.

### Sample preparation for SIRT3 temporal interactions during infection

To determine temporal SIRT3 interactions during the course of infection, small scale IPs were conducted. 2 × 15 cm dishes of SIRT3-GFP and Mito-GFP cells were collected in triplicate at mock, 6, 24, 48, 72, 96, and 120 hpi, and mitochondria isolation kit (Sigma-Aldrich) was used to isolate mitochondria as per protocol. Mitochondria pellets were flash frozen and stored in -80°C. The mitochondria pellets were mechanically lysed by a Polytron with lysis buffer (20 mM HEPES-KOH pH 7.4, 110 mM potassium acetate, 2 mM MgCl_2_, 0.1% Tween-20, 1 M ZnCl_2_, 0.2% Triton X-100, and 1 μM CaCl_2_) containing 1:100 (v/v) protease and phosphatase inhibitor cocktail (Thermo Fisher Scientific, #78446).

Protein A/G magnetic beads (Pierce, #88802) were washed with 1 × DPBS and 2 × lysis buffer, and conjugated to the home-made anti-GFP rabbit antibody for 1 hr at 4°C. The conjugated beads were incubated with mitochondrial lysates at 4°C for 1 hr with gentle rotating. Beads were subsequently washed with 5 × 0.5 mL lysis buffer, followed by one wash in cold MilliQ water. Proteins were eluted with 50 μl TES buffer (106 mM Tris-HCl, 141 mM Tris Base, 2% SDS, 0.5 mM EDTA) for 10 min at 70°C, and concentrated 2.5 times by speed vac. The eluates were reduced and alkylated with 25 mM Tris(2-carboxyethyl)phosphine (TCEP) and 25 mM chloroacetamide for 20 min at 70°C. 2.3 μl 12% phosphoric acid and 165 μl 100 mM TEAB (pH 7.1) in methanol were added to each sample in preparation for S-trap. Samples were subsequently applied to the S-trap columns (Protifi, C02-micro) and washed with 5 × 150 μl 100 mM TEAB pH 7.1 in methanol. Protein samples were digested with trypsin solution (2 μg trypsin in 25 mM TEAB pH 8.0) on column for 1 hr at 47°C. The resulting peptides were eluted sequentially with 40 μl 25 mM TEAB pH 8.0, 0.2% formic acid, and 0.2% formic acid in 50% acetonitrile. The eluted peptides were dried by speed vac and resuspended in 1% formic acid/2% acetonitrile, and subject to mass spectrometry analysis and data processing steps.

### MS analysis of temporal SIRT3 interactions and data processing

Tryptic peptides were applied to an Ultimate 3000 nRSLC system (Dionex) equipped with an EASYSpray ion source (ThermoFisher Scientific). Peptides were separated on an EASY-Spray C18 column (2 μm × 75 μm × 50 cm) using solvents A (0.1% formic acid in water) and B (0.1% formic acid in 97% acetonitrile) with a continuous gradient from 3 to 35% B over 120 min, at a flow rate of 250 nL/min. Eluted peptides were sprayed into a Q Exactive HF mass spectrometer (ThermoFisher Scientific). MS1 and MS2 scans were obtained using a full MS/data dependent MS^2^ mode. MS1 survey scan was performed from 350 to 1800 m/z with 120,000 resolution using an automatic gain control (AGC) of 3E6 and a maximum inject time (MIT) of 50 ms, and the resulting spectra were reported in profile mode. The top 10 most intense precursors were selected for fragmentation by higher energy collision-induced dissociation (HCD) with dynamic exclusion duration of 45 s. The MS2 scans were acquired at a resolution of 30,000 using an AGC target of 1E5, a maximum injection time of 150 ms, an isolation window of 1.6 m/z, a fixed first mass of 100 m/z, a minimum intensity threshold of 1E5, a loop count of 10, dynamic exclusion of 45 s, and spectra were acquired in centroid.

To process the raw spectra files, MS/MS spectra were analyzed by Proteome Discoverer (ver. 2.3, Thermo Fisher Scientific). Mass accuracy was recalibrated by the spectrum files RC node, and the Minora feature detector node was used to detect chromatographic peaks. All MS/MS spectra were searched against a human and herpesvirus protein sequence database (UniProt-SwissProt, downloaded in April 2016) appended with common contaminants (22,241 total entries) using SEQUESST HT node. The following criteria of SEQUEST HT node were applied: full enzyme specificity, maximum missed cleavages of 2, precursor mass tolerance of 5 ppm, fragment mass tolerance of 0.02 Da, static carbamidomethylation of cysteine, and dynamic modifications of methionine oxidation, asparagine deamidation, protein N-terminal Met-loss + acetyl and lysine acetylation. Spectra matches were assessed by Percolator for false discovery rate (FDR) calculation (< 1% FDR) using the reverse sequences of the selected UniProt-SwissProt database.

In the consensus workflow, the feature mapper node was selected for chromatographic retention time alignment between runs with maximum retention time window of 10 min with a 6 ppm mass tolerance. For quantification, peptide abundances were normalized using Total Peptide Amount method, and proteins identified in only one replicate were removed. Imputation was applied using Replicate Based Resampling. A minimum of 2 unique peptides for each protein group and a cutoff of > 1.5 average fold change in any condition was applied. Protein abundances were then imported to Perseus for data imputation using a normal distribution resampling method. Proteins with mitochondrial annotations in GO cellular component were kept, and the protein abundances were normalized to SIRT3 across conditions.

### Quantification of OPA1 K834 acetylation using targeted MS/MS

To acquire precise quantification of acetylation on OPA1 K834 in various contexts, targeted mass spectrometry was performed. 3 × 15 cm dishes of relevant cells were harvested, and mitochondria were enriched using mitochondria isolation kit (Sigma-Aldrich). The mitochondria samples were subject to DDA LC-MS/MS to determine the mitochondrial proteome as previously described, and the abundances of MCU, VDAC3, and CYC1 were used to calculate the normalization factors across samples. PRM assays were carried out by nLC-MS/MS using a Dionex Ultimate 3000 nRSLC coupled to a Q Exactive HF mass spectrometer (Thermo Fisher Scientific). Mitochondrial tryptic peptides were eluted on C18 column by a linear gradient from 0%–35% B over 90 min. The PRM method was set up with the following parameters: profile mode MS2 resolution of 30,000, with AGC target of 1e5, maximum inject time of 300 ms, isolation window of 0.8, and normalized collision energy of 27, and a peptide inclusion list containing the targeted OPA1 peptide GVEVDPSLIK(ac)DTWHQVYR. Summed fragment area normalized by the proteome-derived normalization factors was used for quantification.

### Mitochondrial protein assignment

Previously identified acetylated proteins were searched against human MitoCarta2.0 datasets, and the matched proteins were designated as likely mitochondrial proteins. The protein assignment to distinct mitochondrial compartments was performed using the GO cellular component annotations in UniProt.

### Sample preparation for mitochondrial proteome analysis during infection

Enriched mitochondria pellets were lysed in 2% SDS, 100 mM NaCl, 0.5 mM EDTA, 50 mM Tris, pH 8.2, 1 × Halt protease inhibitor cocktail (Thermo Fisher Scientific), and 100 mg of mitochondrial protein per condition was reduced and alkylated with 25 mM TCEP and 50mM chloroacetamide for 20 min at 70°C, and then subject to methanol/chloroform precipitation. The resulting protein pellets were then dried by speed-vac, and resuspended in 50 mM HEPES, pH 8.2 and digested overnight with trypsin (Promega, Madison) at 37°C (50:1 protein:enzyme w/w ratio). The digested samples were acidified with 1% trifluoroacetic acid (TFA) and mixed on Tomy Shaker for 2 min, and incubated on ice for 15 min, followed by pelleting at 4000 × g for 10 min 4°C. The supernatants were subjected to SDB-RPS StageTip Desalting (3M Analytical Bio-technologies) cleanup per protocol. Peptides were eluted with 50 ul 80% acetonitrile (CAN) and 5% ammonia hydroxide (AmOH) into auto sampler vials. The peptides were dried by speed-vac, resuspended to 2 μg/μL in 1% TFA and 4% CAN, and subjected to DDA LC-MS/MS.

### Analysis of mitochondrial proteome during HCMV infection by mass spectrometry

Mitochondrial samples were lysed in preheated lysis buffer (50mM Tris-HCl pH 8, 100mM NaCl, 0.5mM EDTA, 4% SDS) at 95°C for 5 min. The resulting mitochondrial lysates were reduced and alkylated with 25 mM TCEP and 50 mM chloroacetamide, and incubated at 95°C for 5 min, and subject to methanol/chloroform precipitation method. The protein pellets were then air dried and then resuspended at 0.5 μg/μL by cup horn sonication in 25 mM HEPES pH 8.2. Proteins were incubated with 1:100 trypsin (Promega, Madison) at 37°C with gentle rocking overnight. The digested samples were acidified by 1% TFA with shaking for 2 min and incubated on ice for 15 min, followed by pelleting at 14,000 × g for 5 min. The supernatants were loaded onto a pre-assembled SDB-RPS StageTip Desalting column. Samples were centrifuged at 2,000 × g for 2 min to allow for binding, washed twice with 0.5% TFA, and then eluted with 80% ACN, 5% ammonium hydroxide into auto sampler vials. The resulting samples were dried by speed vac and resuspended in 0.1% formic acid, 2% ACN at a concentration of 2 μg/μL.

MS analysis of the mitochondrial proteome was conducted as described above in the section “MS examination of temporal interactions of SIRT3 during infection”. The abundance ratios over mock calculated by Proteome Discoverer (ver. 2.3, Thermo Fisher Scientific) were used for subsequent analysis.

### Bioinformatics analysis

To generate heatmaps, data were imported into Morpheus (Broad Institute), and K-means clustering was conducted to organize the peptides or proteins. For GO Biological Process analysis, protein names were submitted to Panther database, and a cutoff of FDR ≤ 0.05 was applied, and terms of interest were selected. To generate the SIRT3 interaction network, protein names were imported into Cytoscape (ver. 3.7.0) and analyzed by Reactome Cytoscape plug-in (ver. 6.1.0).

### Acetylation motif analysis

Amino acid sequences of acetyl-peptides were processed by in-house python script and extended by PEPTIDEXTENDER (http://schwartzlab.uconn.edu/pepextend) with Homo sapiens proteome to retrieve the two sequences of 8 amino acids flanking the acetylated lysine (designated as position 0). For visualization, the extended peptides were analyzed by IceLogo (https://iomics.ugent.be/icelogoserver). Percentage difference scoring method was utilized and a p value≤ 0.05 was set as a cutoff, with Homo sapiens proteome database as the reference set.

### Fuzzy c-means clustering and GO enrichment analysis

Clustering of the temporal abundances of acetylated peptides were performed by the fuzzy c-means clustering using SciKit-Fuzzy (v0.4) python package. Fuzzy c-means parameter m and error were set as 2 and 0.005, respectively. To perform GO enrichment analysis, protein UniProt accession numbers corresponding to the acetylated peptides from each cluster were submitted to Panther (http://pantherdb.org/) to perform statistical overrepresentation test for GO biological processes, and representative terms (FDR≤ 0.05) were selected from the resulting list.

### Transient expression of OPA1 constructs

pMSCV-OPA1 (Plasmid #26047) was purchased from Addgene. To generate OPA1 K931R and K931Q mutant constructs, site-directed mutagenesis was conducted using QuickChange II Site-Directed Mutagenesis kit as per instruction (Agilent). Primers used for the mutagenesis are as follows: OPA1K931R: 5’- CCAGTTGAACGCGTCTACCAGTAAGCAATTTAATCTTCTTCT– 3’ and 5’–AGAAGAAGATTAAATTGCTTACTGGTAGACGCGTTCAACTGG– 3’. OPA1K931Q: 5’—CCGCCAGTTGAACGCGTTGACCAGTAAGCAATTTAAT—3’ and 5’–ATTAAATTGCTTACTGGTCAACGCGTTCAACTGGCGG– 3’. Constructs were selected by ampicillin resistance and validated by sequencing. At 60% confluence, MRC5 cells on a 6-well plate were transfected with 1 μg of the OPA1 construct using Lipofectamine 2000 for 12 hours as per manual (Thermo Fisher Scientific). pCMV-VSV-G (Plasmid #8454) was used as the over-expression control.

### siRNA-mediated knockdowns

siRNA-mediated knockdowns were carried out by transfecting MRC5 cells with siRNAs of SIRT3 (Dharmacon J-004827-11-0002, and J-004827-12-0002), ACAA2 (Dharmacon J-009408-05), or OPA1 (Dharmacon LU-005273-05, LU-005273-06, LU-005273-07, LU-005273-08). At 60% confluence, MRC5 cells on a 12-well plate were transfected with 40 pmol of the targeting siRNAs or control siRNA (Sigma-Aldrich, SIC006) using Lipofectamine RNAimax as per protocol (Thermo Fisher Scientific). To validate the efficacy of knockdowns, cells were collected at 72 h after transfection, and subject to western blotting. To assess the effect of knockdowns on HCMV production, transfected cells were infected with HCMV at MOI of 3 at 72 h after transfection, and the resulting supernatants at 120 hpi were analyzed to viral titer assays.

### Microscopy analyses

Microscopy was performed using a Leica TC SP5 confocal microscope (Leica Microsystems) equipped with a 63× oil-immersion objective lens. Live cells were treated with MitoTracker Red CMXRos prior to fixation to visualize mitochondria. To visualize viral proteins, cells were washed twice with DPBS, and then fixed in 4% PFA at room temperature for 15 mm. The fixed cells were washed with 1 mL DPBS three times, and permeabilized with DPBS containing 0.2% Tween-20 (PBST) for 10 min at room temperature, and blocked with blocking buffer (5% human serum, 3% bovine serum albumin (BSA), PBST) for 1 hr at room temperature. Samples were subsequently incubated with primary antibodies against IE1 (gift from Thomas Shenk, clone 1B12, 1:150) or pUL99 (gift from Thomas Shenk, clone 10B4, 1:150) for 1 hr in 3% BSA in 50 mM Tris Based Saline Tris-Cl, pH 7.5. 150 mM NaCl with 0.1% Tween-20 (TBST). Samples were then washed twice with TBST and treated with secondary Alexa Fluor 555 conjugated goat antibody (AB_141780, Life Technologies, 1:1000) diluted in 3% BSA in 0.1% TBST. Cells were washed twice, followed by treatment with Hoechst 33258 (1:10000) in 0.1% TBST for 15 min at room temperature. To assess mitochondrial morphology, cells cultured on 35 mm glass-bottom scope dishes (MatTek) were stained with 50 nM MitoTracker Red CMXRos (Cell Signaling technology, #9082) for 15 min and washed with culture medium twice. To measure mitochondrial area, >30 mitochondria were selected at the periphery of each cell, and the area was measured by ImageJ. Mitochondrial morphology analyses were conducted using MitoMorph (https://academic.oup.com/nar/article/48/2/605/5651332) plugin in ImageJ. Parameters used for the classification of objects are as follows. Filament network: total length > 11 μm, or total length > 5 μm and minimum linear extension > 1.5 μm and solidity < 0.6; fragment / puncta: area < 1.2 μm^2^, minimum linear extension < 1.2 μm, circularity > 0.5, and aspect ratio < 3; swollen: area > 1.2 μm^2^, minimum linear extension > 1.1 μm, total length < 5 μm, circularity > 0.5, aspect ratio < 4, solidity > 0.6; rods / intermediate: all the objects not satisfying any of the above criteria. Each biological replicate embodies ≥ 9 images of multiple cells (n>60, 70 or 75, as indicated in the figure legends), and averaged percentages of distinct mitochondrial morphology were calculated for each biological replicate. Three biological replicates were used for each condition and statistical analyses were conducted using student-t test.

### Viral titer assay by IE1 staining

MRC5 cells and cell lines were infected with HCMV at the indicated MOIs, and supernatants were collected at 120 hpi. The supernatants were serially diluted and applied to a reporter plate of wild-type MRC5 cells. After 24 h, the infected cells were washed 3 x with cold PBS and fixed with cold methanol for 20 min at -20°C, blocked with blocking buffer (3% BSA 0.2% Tween-20 in PBS). Samples were then treated with anti-IE1 antibody (a gift from the Shenk lab, 1:100)for 1 hr at room temperature, followed by 3 × TBST washes and 1 hr treatment with secondary antibody Alexa 488 anti-mouse goat IgG (1:1000; Life Technologies, A-11001). Samples were subsequently stained with Hoechst 33258 (1:1000) for 15 min at room temperature. Viral titers were calculated with the Operetta imaging system (Perkin Elmer). Three biological replicates were conducted for each condition.

### Viral titer assay by TCID50

MRC5 cells and cell lines were infected with HCMV at the indicated MOIs, and supernatants were collected at 120 hpi. Ten-fold serial dilutions of the supernatants were made in DMEM complete growth medium, and applied to a wild-type MRC5 cells in a 96-well plate in triplicates. 12 days after infection, the 50% tissue culture infectious dose (TCID50) was calculated according to the method of Reed and Muench [92]. Three biological replicates were conducted for each condition.

### Western blot analysis

Collected cells were lysed in 1x Laemlli sample buffer (62.5 mM Tris-HCL, pH 6.8, 2% SDS (w/v), 10% glycerol (v/v), 0.02% bromophenol blue (w/v), 100 mM DTT) and boiled at 95°C for 10 minutes. Protein concentrations were determined by Pierce BCA test (Fisher Scientific), and equal amounts of proteins were loaded on a 10% Tris-glycine SDS-polyacrylamide gel at 130 V for 1.5 h. The proteins on gel were transferred onto a PVDF membrane (Bio-Rad) overnight at 4°C at 35 V by a wet transfer method. Membranes were then blocked in blocking buffer (5% milk (w/v), 0.2% TBST) for 1 hr and incubated with indicated primary antibodies over-night at 4°C. Membrane was washed with 3 × TBST, followed by incubation with fluorescently-tagged secondary antibodies (IRDye 680nm and 800nm) for 30 min. Immunofluorescence detection and analyses was performed using the LI-COR Odyssey CLx imaging system.

Primary antibodies used for western blot are: GFP (AB_390913, Roche, 1:5000);

OPA1 (AB_2734117, Cell Signaling Technology, 1:500); SIRT3 (AB_10861832, Abcam, 1:400); tubulin (AB_1965960, 1:5000); ACAA2 (AB_2635746, 1:500); CMV pUL44 (ICP36) (CA006-100, Virusys, 1:1000); IE1 (gift from Thomas Shenk, clone 1B12, 1:400); IE2 (gift from Thomas Shenk, 3H9, 1:400); pUL26 (gift from Thomas Shenk, 7H1-5, 1:400); pUL99 (gift from Thomas Shenk, clone 10B4, 1:150).

### Cell viability assay by Trypan Blue

To assess cell viability, cells of interest were trypsinized and pelleted, and resuspended in culture medium. 10 ul of cell suspension was mixed with equal volume of 0.4% Trypan Blue dye by pipetting up and down. Mixed cells were mounted to a hemacytometer counter. Stained (dead) and unstained (alive) cells were counted and the percentage of viable cells was calculated. 3 technical replicates were performed per condition.

### Measurement of mitochondrial pH and mitochondrial membrane potential

For mitochondrial membrane potential measurement, cells were incubated with 2 μM JC-1 (M34152, ThermoFisher Scientific) at 37°C for 10 min. Cells were washed twice with DPBS, and kept in fresh culture medium. Confocal images were taken using Leica SP5 platform with a 488 nm argon excitation laser and emission wavelengths of 527 nm (green) and 590 nm (red). The average red to green fluorescence intensity ratio of images was used as the measurement of mitochondrial membrane potential.

To measure mitochondrial pH, cells on 35 mm glass-bottom scope dishes were transfected with 500 ng mito-SypHer (#48251, Addgene, 21224385). 48 hrs after treatment, cells were treated as indicated, and confocal images were taken at indicated time points. Cells were excited at 405/480 nm with 505 ~ 535 nm emission filter. The average 480 to 405 nm fluorescence ratio was a measurement of mitochondrial pH. To calibrate pH measurement, MRC5 cells transfected with mito-SypHer were washed twice with DPBS, and then fixed and permeabilized with -20°C methanol for 20 min. Fixed cells were treated with DPBS with a pH range of 7.0 ~ 8.2, and the resulting 488/405 fluorescence ratios were fitted to generate the standard curve.

### Statistical analyses

Statistical analyses were conducted using a two-tailed student’s t test unless otherwise stated. Data are presented as mean ± SD or mean ± SEM as described in the figure legends. Numbers of biological and technical replicates are indicated in figures and respective figure legends. Data meet the requirements of the tests used.

## Supporting information

S1 Fig Characterizing the temporal mitochondrial acetylome during HCMV infection. A. Average relative peptide acetylation levels in different subcellular localizations. The average abundances of the acetylated peptides at different time points of infection are normalized to the corresponding protein abundances. B. Frequency density of normalized acetylated peptides abundances (log2 fold-change) in the mitochondria and non-mitochondrial localizations at 120 hpi. The acetylated peptide abundances were normalized to the corresponding protein abundances. C. Heatmap of normalized protein abundances in distinct mitochondrial compartments during HCMV infection. D. Motif sequence patterns for mitochondrial acetylated peptides identified at different stages of HCMV infection. The numbers of acetylated peptides are shown at the top for each time point. E. Normalized acetylated peptide abundances clustered by Fuzzy c-means. Membership scores indicate the confidence of the cluster assignment. The motif patterns and the enriched GO biological processes in each cluster are displayed under each corresponding cluster.
(TIF)

S2 Fig SIRT3 enzymatic activity is critical for its antiviral effect. A. TCID50 titers of cells over-expressing mito-GFP, SIRT3, or SIRT3-H248Y mutant. TCID50 assays were conducted using the supernatants collected at 120 hpi in triplicates. * p-value < 0.05, *** p-value < 0.005. B. Viral protein abundances during infection in the context of SIRT3 WT or mutant over-expression. Protein abundances measured by densitometry were normalized to respective tubulin control, and the mutant/WT protein abundance ratios are indicated below the bands. C. Protein abundance of SIRT3 during viral infection in cells with siRNA-mediated knockdown. The relative protein abundance ratios of SIRT3 (normalized to tubulin) upon siRNA knockdown are indicated below the respective bands.
(TIF)

S3 Fig Acetylation levels normalized to protein abundances for SIRT3 substrates during HCMV infection. A. Venn diagram of proteins identified in the acetylome dataset, characterized as human SIRT3 substrates, and putative SIRT3 substrates derived from multiple mouse Sirt3-KO studies. B. Normalized acetylated peptide abundances of putative SIRT3 substrates during HCMV infection. Acetylated peptides are grouped by their associated proteins, and color-coded by their abundances normalized to protein abundances (log2 fold changes).
(TIF)

S4 Fig Regulation of OPA1 impacts mitochondrial fission-fusion balance during infection. A. Confirmation of OPA1 over-expression and siRNA-mediated knockdown by western-blot. One representative construct (#2) was chosen for OPA1 siRNA knockdown. Protein abundances measured by densitometry were normalized to respective tubulin control, and the mutant/WT protein abundance ratios were generated and indicated below the bands. B. Cell viability during the treatment of OPA1 siRNA knockdown and over-expression. Cell death percentages were measured using Trypan blue, and 2% hydrogen peroxide was used as the positive control. n = 3 technical replicates for each group. C. Mitochondrial morphology percentages of cells with siOPA1 knockdown at mock, 24, and 72 hpi. Mitochondrial area was calculated based on MitoTracker Red CMXRos signal. Student t-test was conducted to determine the differences in fragment between mock and infected groups. * p-value < 0.05, ** p-value < 0.01, *** p-value < 0.005. Three biological replicates were used for each group (n > 70 cells per replicate). D-E. WB of OPA1 and viral protein abundances during HCMV infection in cells over-expressing OPA1 constructs. Exposure was lower than the same western blots shown in Fig 4E. Immediate early viral markers: IE1 and IE2; early: pUL26 and pUL44; late: pUL99. Densities of OPA1 and viral proteins were normalized to tubulin, and then divided by the normalized abundance of respective time point in siControl (E). F. Protein abundance of OPA1 WT and mutants during HCMV infection. G. Mitochondrial morphology percentages of cells over-expressing OPA1 constructs at at mock, 24, and 72 hpi. Mitochondrial area was calculated based on MitoTracker Red CMXRos signal. Student t-test was conducted to determine the statistical differences in fragment between groups. * p-value < 0.05, ** p-value < 0.01, *** p-value < 0.005, **** p-value < 0.001. Three biological replicates were used for each group (n > 70 cells per replicate).
(TIF)

S1 Table Mitochondrial proteome during the course of HCMV infection. (XLSX)

S2 Table Mitochondrial acetylome grouped by mitochondrial compartments. (XLSX)

S3 Table Acetylation abundances of characterized and putative SIRT3 substrates. (XLSX)

S4 Table Specific SIRT3 interactions at 72 hpi. (XLSX)

S5 Table Measurements of mitochondrial morphology. (XLSX)

S6 Table Temporal SIRT3 interactions overlaid with mitochondrial acetylome and proteome. (XLSX)
