# Supplementary figures and images for: The antiviral sirtuin 3 bridges protein acetylation to mitochondrial integrity and metabolism during human cytomegalovirus infection
Source: PLoS Pathog. 2021 Apr 15;17(4):e1009506. doi: 10.1371/journal.ppat.1009506 (PMC8078788; doi:10.1371/journal.ppat.1009506)

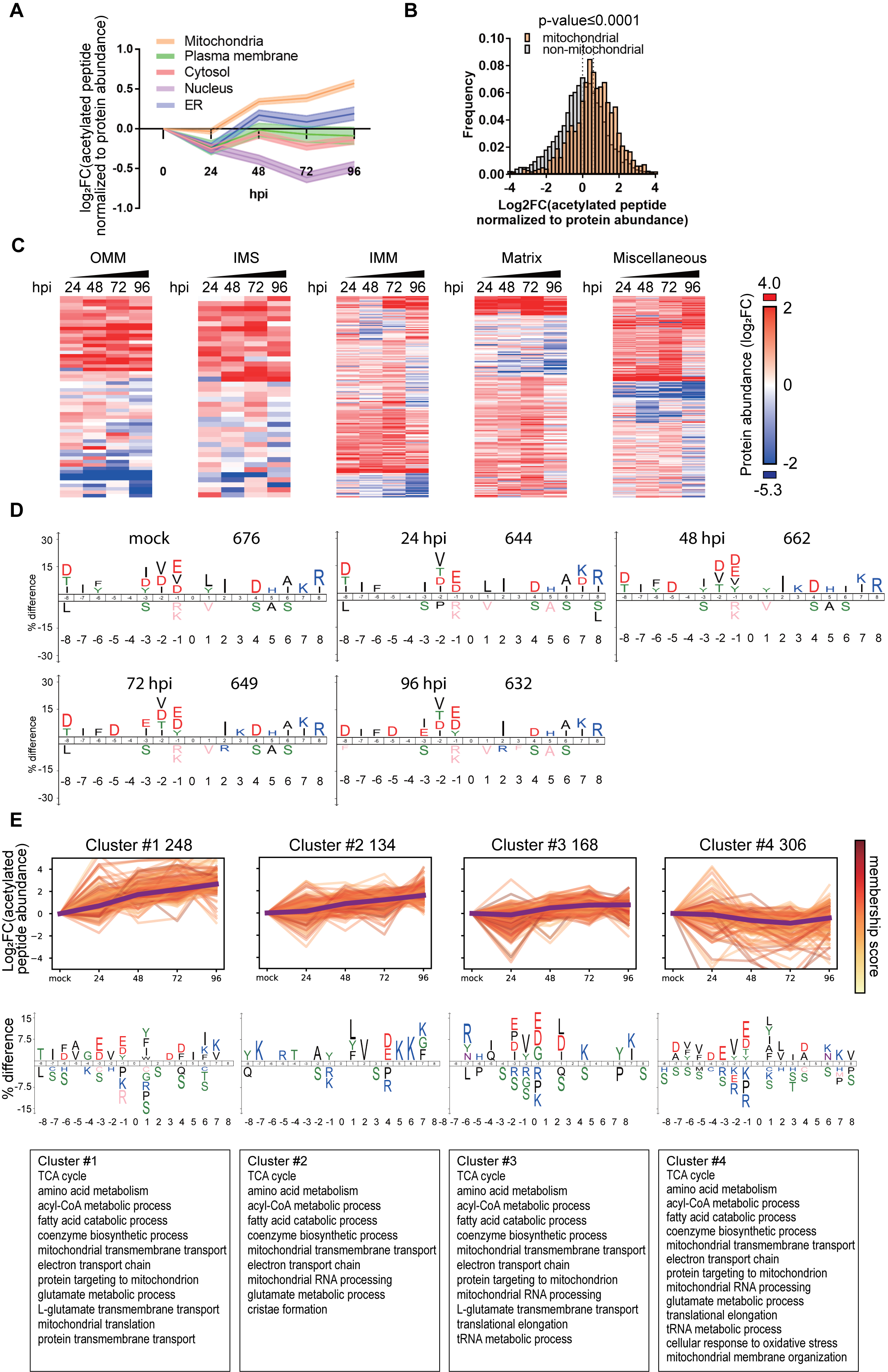

Supplement: S1 Fig — A. Average relative peptide acetylation levels in different subcellular localizations. The average abundances of the acetylated peptides at different time points of infection are normalized to the corresponding protein abundances. B. Frequency density of normalized acetylated peptides abundances (log2 fold-change) in the mitochondria and non-mitochondrial localizations at 120 hpi. The acetylated peptide abundances were normalized to the corresponding protein abundances. C. Heatmap of normalized protein abundances in distinct mitochondrial compartments during HCMV infection. D. Motif sequence patterns for mitochondrial acetylated peptides identified at different stages of HCMV infection. The numbers of acetylated peptides are shown at the top for each time point. E. Normalized acetylated peptide abundances clustered by Fuzzy c-means. Membership scores indicate the confidence of the cluster assignment. The motif patterns and the enriched GO biological processes in each cluster are displayed under each corresponding cluster. (TIF) [file ppat.1009506.s001.tif]

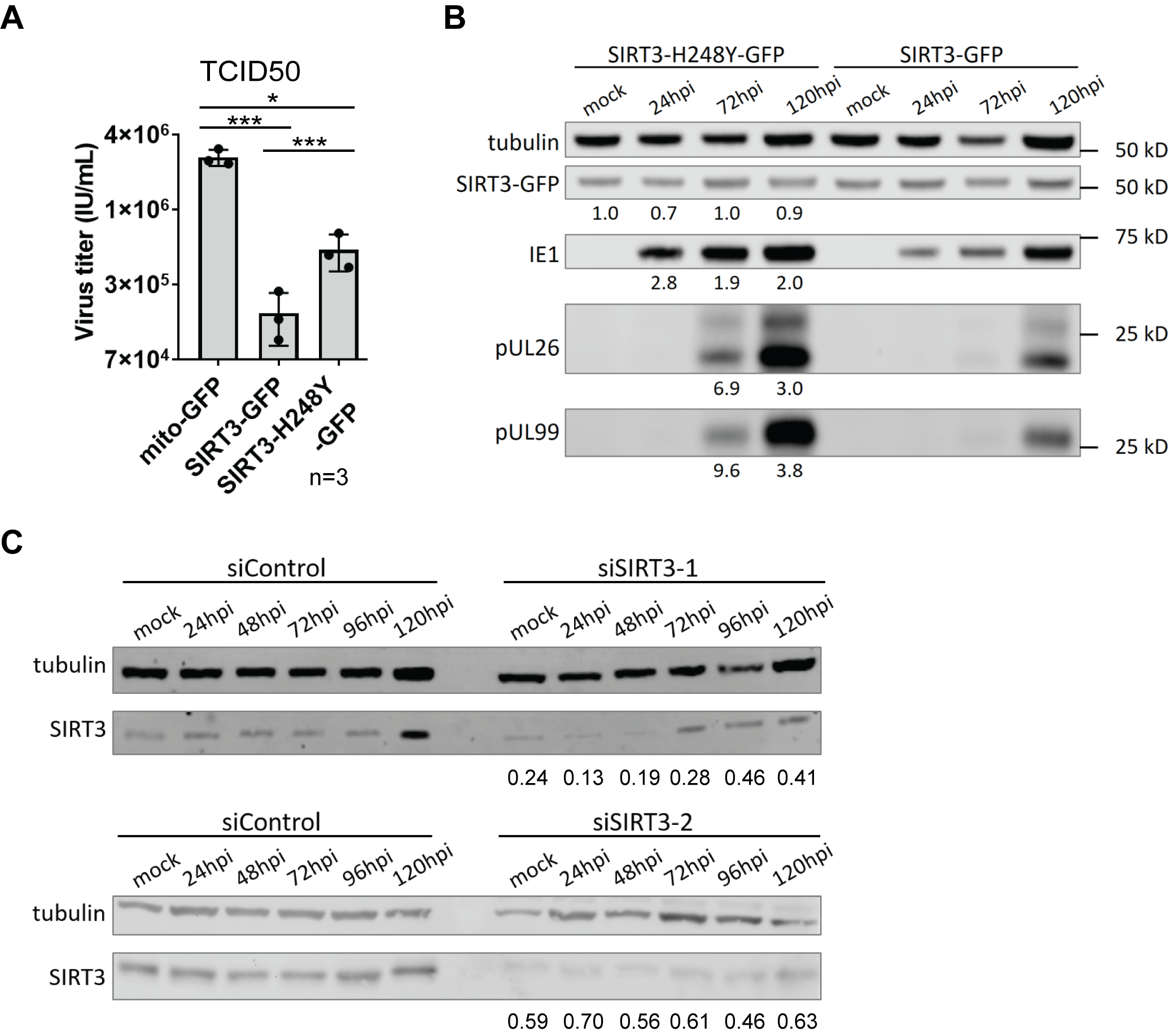

Supplement: S2 Fig — A. TCID50 titers of cells over-expressing mito-GFP, SIRT3, or SIRT3-H248Y mutant. TCID50 assays were conducted using the supernatants collected at 120 hpi in triplicates. * p-value < 0.05, *** p-value < 0.005. B. Viral protein abundances during infection in the context of SIRT3 WT or mutant over-expression. Protein abundances measured by densitometry were normalized to respective tubulin control, and the mutant/WT protein abundance ratios are indicated below the bands. C. Protein abundance of SIRT3 during viral infection in cells with siRNA-mediated knockdown. The relative protein abundance ratios of SIRT3 (normalized to tubulin) upon siRNA knockdown are indicated below the respective bands. (TIF) [file ppat.1009506.s002.tif]

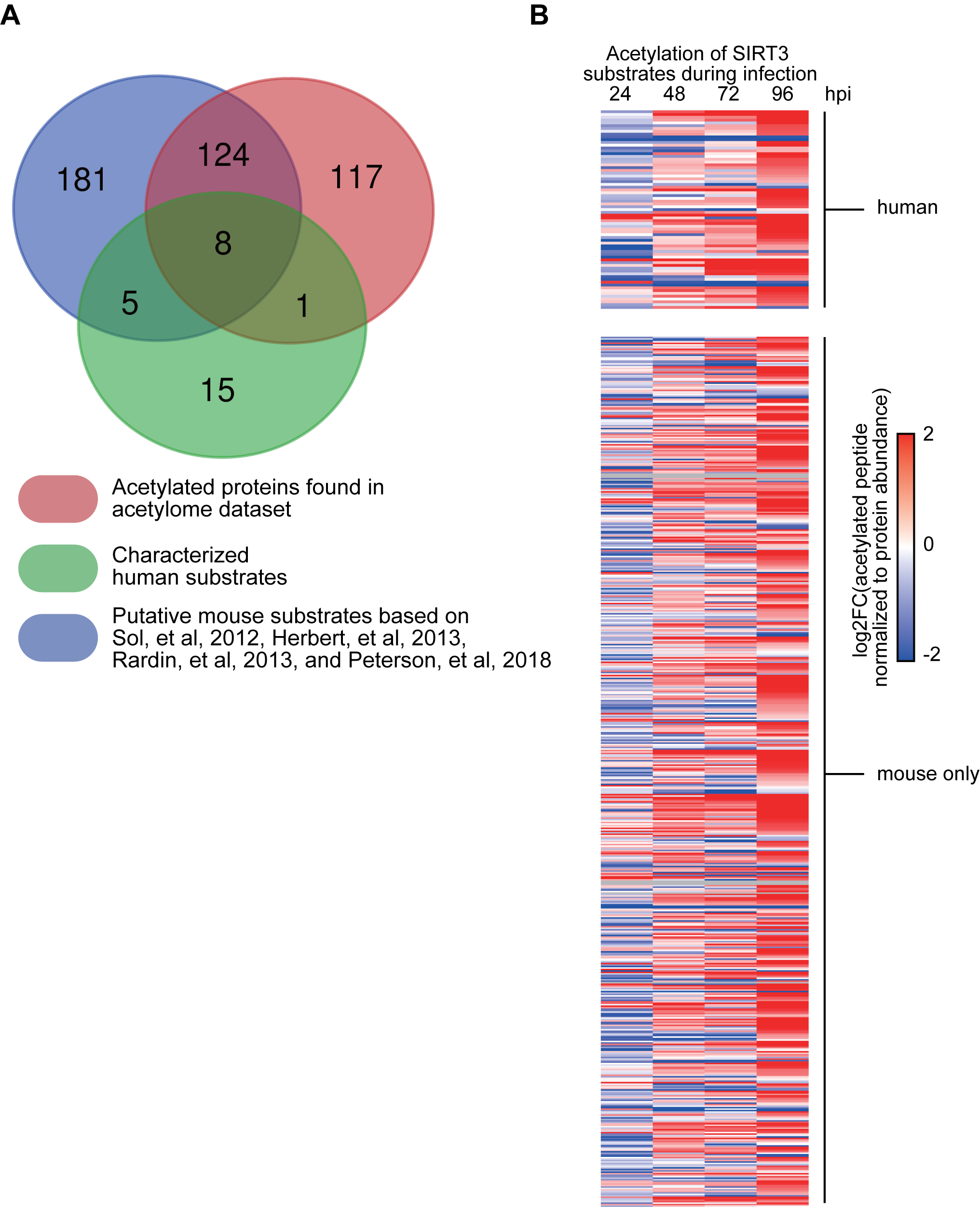

Supplement: S3 Fig — A. Venn diagram of proteins identified in the acetylome dataset, characterized as human SIRT3 substrates, and putative SIRT3 substrates derived from multiple mouse Sirt3-KO studies. B. Normalized acetylated peptide abundances of putative SIRT3 substrates during HCMV infection. Acetylated peptides are grouped by their associated proteins, and color-coded by their abundances normalized to protein abundances (log2 fold changes). (TIF) [file ppat.1009506.s003.tif]

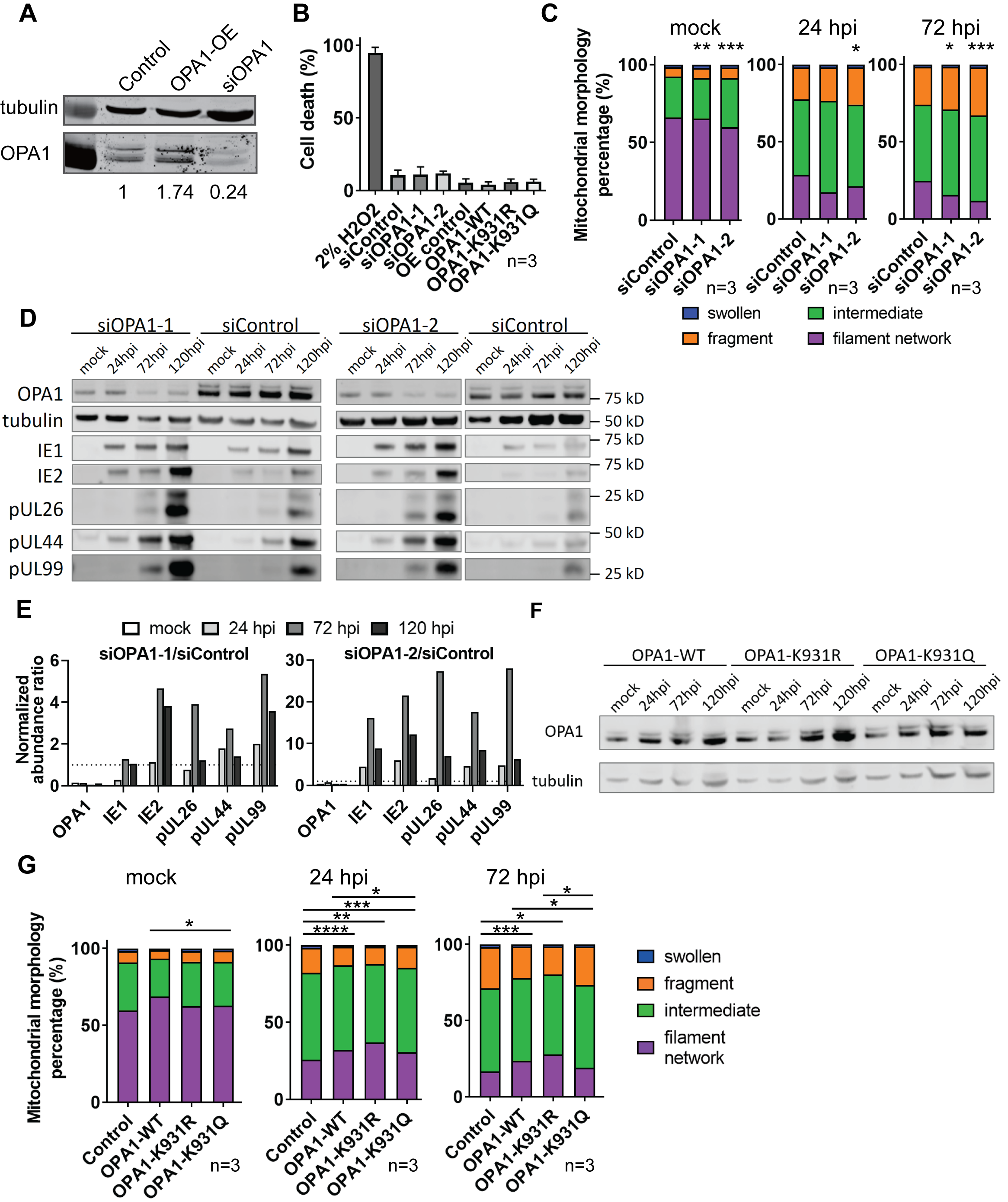

Supplement: S4 Fig — A. Confirmation of OPA1 over-expression and siRNA-mediated knockdown by western-blot. One representative construct (#2) was chosen for OPA1 siRNA knockdown. Protein abundances measured by densitometry were normalized to respective tubulin control, and the mutant/WT protein abundance ratios were generated and indicated below the bands. B. Cell viability during the treatment of OPA1 siRNA knockdown and over-expression. Cell death percentages were measured using Trypan blue, and 2% hydrogen peroxide was used as the positive control. n = 3 technical replicates for each group. C. Mitochondrial morphology percentages of cells with siOPA1 knockdown at mock, 24, and 72 hpi. Mitochondrial area was calculated based on MitoTracker Red CMXRos signal. Student t-test was conducted to determine the differences in fragment between mock and infected groups. * p-value < 0.05, ** p-value < 0.01, *** p-value < 0.005. Three biological replicates were used for each group (n > 70 cells per replicate). D-E. WB of OPA1 and viral protein abundances during HCMV infection in cells over-expressing OPA1 constructs. Exposure was lower than the same western blots shown in Fig 4E. Immediate early viral markers: IE1 and IE2; early: pUL26 and pUL44; late: pUL99. Densities of OPA1 and viral proteins were normalized to tubulin, and then divided by the normalized abundance of respective time point in siControl (E). F. Protein abundance of OPA1 WT and mutants during HCMV infection. G. Mitochondrial morphology percentages of cells over-expressing OPA1 constructs at at mock, 24, and 72 hpi. Mitochondrial area was calculated based on MitoTracker Red CMXRos signal. Student t-test was conducted to determine the statistical differences in fragment between groups. * p-value < 0.05, ** p-value < 0.01, *** p-value < 0.005, **** p-value < 0.001. Three biological replicates were used for each group (n > 70 cells per replicate). (TIF) [file ppat.1009506.s004.tif]
